# Interpreting elevated liver blood test results through a genetic lens: A genome‐wide association study

**DOI:** 10.1111/liv.16114

**Published:** 2024-10-19

**Authors:** Hamish Innes, Stephan Buch, Timothy J. Kendall, Jonathan A. Fallowfield, Indra Neil Guha

**Affiliations:** ^1^ School of Health and Life Sciences Glasgow Caledonian University Glasgow UK; ^2^ BBV/STI team, Public Health Scotland Glasgow UK; ^3^ Lifespan and Population Health University of Nottingham Nottingham UK; ^4^ Medical Department 1 University Hospital Dresden Dresden TU Germany; ^5^ Centre for Inflammation Research, Institute for Regeneration and Repair University of Edinburgh Edinburgh UK; ^6^ Edinburgh Pathology University of Edinburgh Edinburgh UK; ^7^ NIHR Nottingham Biomedical Research Centre Nottingham University Hospitals NHS Trust and the University of Nottingham Nottingham UK; ^8^ Nottingham Digestive Diseases Centre, School of Medicine University of Nottingham Nottingham UK

**Keywords:** chronic liver disease, genetics, personalized medicine, prognosis, risk stratification

## Abstract

**Background and Aims:**

Individuals with genetic polymorphisms in *UGT1A1* exhibit bilirubin levels that belie their risk of liver disease (Gilbert's syndrome) but it is not known if this phenomenon extends to other common liver blood tests (LBTs).

**Methods:**

A genome‐wide association analysis of 10 LBTs was conducted using the UK biobank. Polygenic scores (PGS) were created from discordant loci (e.g. loci associated with the LBT but *not* associated with cirrhosis morbidity risk). Participants were assigned to a low, intermediate or high PGS for each LBT. A high PGS approximates Gilbert's syndrome (i.e. elevated LBT without an analogous increase in disease risk). The prognostic significance of an ‘elevated’ LBT—and how this differs by PGS—was assessed through competing risk survival analysis.

**Results:**

This study included 157 005 and 166 871 participants for the discovery and validation phases, respectively. Elevated LBTs were more prevalent in the high versus low PGS group, yet the 10‐year risk of cirrhosis morbidity was comparable. For example, in the low PGS group, 4.3% had an elevated gamma‐glutamyltransferase (GGT) and the 10‐year risk of cirrhosis morbidity was .45%. Conversely, in the high PGS group, 21.2% had an elevated GGT and the 10‐year risk was .38%. Accordingly, the 10‐year risk of cirrhosis morbidity for individuals with an elevated GGT was markedly different in the low vs. high group (4.2% vs. 1.2%; *p* < .001). Similar results were apparent for Fibrosis‐4 index, total bilirubin, and platelet count.

**Conclusion:**

Variability in LBTs is influenced by genetic polymorphisms that have a neutral effect on disease risk. These findings have implications for interpreting elevated LBTs in clinical practice.

**Key points**

- It is not known if the concept of Gilbert’s syndrome applies to liver blood tests (LBTs) beyond bilirubin and what implications this may have for personalized medicine.
- The prognostic significance of an elevated LBT can vary considerably depending on an individual’s genetics.
- Differences were particularly pronounced in relation to gamma‐glutamyl transferase, Fibrosis‐4 index, platelet count as well as total bilirubin.
- The relevance of this phenomenon in other areas of medicine is unclear and not well explored.

## 1 INTRODUCTION

Targeting clinical interventions and monitoring strategies in people with elevated biomarker(s) is commonplace in medicine and is intrinsic to risk stratification and resource allocation. Inherently, this approach assumes that individuals with elevated biomarker levels have a higher risk of the disease of interest than those with ‘normal’ values. In reality, biomarker values are influenced by a variety of covariates (such as age, ethnicity, and sample collection conditions), not just the presence/severity of a given disease.^1^ One notable source of biomarker variability that is underexplored (and therefore not taken into account) is germline genetics. Of particular relevance to personalized medicine is the degree to which biomarker values are modified by genetic factors that are neutral to (i.e. not associated with) the disease of interest.^1^ Where this is the case, the definition of an ‘elevated’ biomarker may vary from one individual to the next, which complicates clinical interpretation.

Recently, genetic adjustment of prostate‐specific antigen (PSA), a widely used biomarker for prostate cancer, was proposed to account for non‐cancer‐related variation in PSA and improve clinical decision‐making.^2^ However, a longer‐established clinical example is total bilirubin, which is a biomarker/indicator of chronic liver disease (CLD).^3^ Individuals with Gilbert's syndrome (GS) exhibit genetic polymorphisms in the region of uridine diphosphate glucuronosyltransferase 1A1 (*UGT1A1*), causing a 60%–70% reduction in the liver's ability to conjugate bilirubin, and thus leading to substantially higher serum bilirubin levels.^4^ Despite their higher bilirubin levels, individuals with GS are at no greater risk of developing cirrhosis or liver‐related morbidity. The interpretation of an elevated bilirubin level in relation to liver disease prognosis is fundamentally different, therefore, for individuals with GS versus those without. For example, for individuals without GS, an elevated bilirubin is likely to reflect CLD, and as such, the 10‐year risk of liver disease morbidity/mortality will be high. Conversely, for individuals with GS, an elevated bilirubin is more likely to reflect the individuals' high‐normal, and the 10‐year of liver morbidity/mortality will be lower.

In this study, we set out to understand if the concept of GS applies to liver blood tests (LBTs) beyond bilirubin and what implications this may have for personalized medicine strategies. LBTs such as transaminases are widely used by healthcare systems to identify and monitor people with CLD. Population studies have shown that over a 10‐year period, approximately 25% of an entire healthcare catchment area will have liver enzymes measured, 20% of these tests will be abnormal at some point.^5^, ^6^ While a proportion of these test abnormalities represent CLD, a significant number do not.^7^ As importantly, emerging studies over the last 10 years highlight the burden of liver disease in those with risk factors but normal liver enzyme tests.^8^, ^9^ Understanding the role of genetic variability and its relevance to LBTs beyond bilirubin may allow clinicians to distinguish an elevated LBT reflecting serious liver disease from an elevated LBT that merely reflects a patient's high baseline. Thus, the goal of this study was to systematically explore baseline variability in standard LBTs and assess the implications for CLD risk stratification.

## 2 METHODS

### 2.1 Data source

UK Biobank (UKB) is a community cohort study of more than half a million individuals in the UK (*n* = 502 492). Participants were interviewed from May 2006 to July 2010 at 22 UKB assessment centres located throughout the UK. All individuals aged 40–69 years and living within 25 miles of an assessment centre (approximately 9 million people in total) were sent an invitation letter for the study. During the interview, participants completed a comprehensive health questionnaire, a physical examination, and donated biological specimens. Follow‐up data on subsequent health outcome events are supplied through record linkage to UK mortality, hospital admission, and cancer registries.^10^

LBT values were derived from blood specimens collected at UKB enrolment. The laboratory assays/methods used by UKB to generate these data have been described in detail.^11^, ^12^ Version 3 of the UKB genetic dataset, providing array‐based genetic sequencing data for *n* = 487 320 participants, was used throughout. Individuals with poor‐quality genetic data—defined via extreme levels of heterozygosity (Field ID: 22027)—were omitted from this dataset.

UKB has approval from the UK North West multi‐centre Research Ethics Committee. Informed written consent was obtained from each participant.

### 2.2 Participant exclusion criteria

All participants were initially eligible for inclusion in the study, but we excluded those who were (a) not included in the UKB genetic dataset; (b) related to another participant (defined as a kinship coefficient >.1); or (c) missing LBT data.

In addition, we excluded individuals with prevalent cirrhosis morbidity, defined as a cirrhosis‐related hospital admission before UKB enrolment. For simplicity, and to guard against confounding by population stratification, we further excluded individuals who were not of White British ancestry (defined by the core UKB team: Field ID: 22006).

### 2.3 Discovery and validation subgroups

In broad terms, the discovery group was used to derive polygenic scores (PGS), whereas the validation group was used to assess their clinical relevance in terms of estimating 10‐year risk.

The discovery cohort included the latter 50% of UKB participants (enrolment date: Feb 2009 to Oct 2010). Conversely, the first 50% of participants were included in the validation subset (enrolment date: April 2006 to Jan 2009). This order was chosen to maximize the number of incident cirrhosis morbidity cases in the validation group, in order to model 10‐year risk as precisely as possible.

### 2.4 Liver blood tests

We considered seven simple LBTs: total bilirubin, ALT; albumin, alkaline phosphatase (ALP), aspartate aminotransferase (AST); gamma‐glutamyl transferase (GGT); and platelet count (PC).

In addition, we included three multivariable LBTs: Aspartate aminotransferase platelet ratio index (APRI), Fibrosis‐4 index (FIB‐4), and the Forns index; three of the most extensively validated non‐invasive panel tests comprising routinely collected variables. Thus, 10 LBTs were included in total.

### 2.5 Genome‐wide association analyses

For quality control, we excluded variants from the UKB genetic dataset meeting any of the following criteria: (a) minor allele frequency <1%, (b) imputation information score <.8; (c) gross deviation from Hardy Weinberg equilibrium (*p* < 1.0 × 10^−7^); (d) extreme level of missingness (>10%); and (e) non‐biallelic or duplicate variants.

All 10 LBT phenotypes were log‐transformed to achieve approximate normality. A linear regression genome‐wide association analysis was then undertaken for each log‐transformed LBT (i.e. with log‐transformed LBT as the dependent variable). This genome‐wide analysis included participants in the discovery analysis cohort only. All associations were adjusted for age, sex, and the first 10 principal components of genetic ancestry.

To identify a set of loci independently associated with the LBT, we first selected loci associated at *p*‐value < 5.0 × 10^−8^. A standard clumping procedure was then performed to identify lead loci (using distance parameter 1000 KB, *r* ^2^ parameter = <.05).^13^

In broad terms, each lead locus will exhibit three characteristics:

- Association with the corresponding LBT at *p* < 5.0 × 10^−8^.
- A lower *p*‐value than all other loci in the nearby genomic region.
- No correlation with (i.e. statistically independent from) other lead loci.

### 2.6 Identifying discordant lead loci

For each LBT, lead loci identified from the clumping procedure were assigned to one of two mutually exclusive groups:

Group A loci: A variant was defined as a group A locus if it exhibited a statistically significant association with cirrhosis morbidity risk that was directionally concordant to its association with the LBT. For instance, suppose the well‐known rs738409 variant in *PNPLA3* was identified as a lead locus for FIB‐4—in this scenario, if the rs738409:G allele was associated with higher FIB‐4 values, then rs738409 would be considered a group A locus for FIB‐4 if the rs738409:G allele was also significantly associated with a higher risk of cirrhosis morbidity. This is because a higher FIB‐4 value indicates a higher risk of disease; thus, the two associations are directionally concordant.
Group B loci: Group B loci comprises all lead loci whose association with the LBT does not mirror the association with disease (i.e. cirrhosis morbidity). For example, suppose the well‐known rs429358:C locus in *APOE* was associated with a higher FIB‐4 score. If this same variant was *not* associated with a higher risk of cirrhosis morbidity, then rs429358:C would be considered a group B locus. According to the GS paradigm, group B loci create a disconnect between an individual's LBT value and their true risk of disease. Taking account of this disconnect may help to improve the interpretation of an elevated LBT.

Association with cirrhosis morbidity risk was determined from a time‐to‐event Cox regression analysis. Participants were followed from the date of UKB enrolment through to the earliest of: (a) date of death (if at all); (b) registry completion; or (c) date of cirrhosis morbidity (if at all). The outcome event—cirrhosis morbidity—was defined using a standard set of ICD codes (Table S1).

One‐tail *p*‐values were calculated, considering the directional concordance between each locus's association with: (a) the LBT; and (b) risk of cirrhosis morbidity.

To correct for multiple testing, a conservative false discovery rate (FDR) threshold <10% was used to define statistical significance. Thus, lead loci associated with cirrhosis morbidity at FDR <10% were assigned to group A; all other lead loci were assigned to group B.

### 2.7 Polygenic score development

A PGS was calculated for each LBT, using group B loci only. Because PGSs were derived from group B loci only, our a priori hypothesis was that higher PGS values would be associated with the LBT value, but not with the risk of disease. Thus, the PGS is intended to inform how the clinical significance of an elevated LBT value is interpreted.

All PGSs were calculated using the plink 2.0 software package, according to the formula:

$$\mathrm{PGS}\;=\;\sum_{i=1}^{k}w_{i}X_{i}$$

where *k* is the number of independent group B loci, *w*_*i*_ is the effect size (i.e. beta) of each variant with the LBT; *X*_*i*_ is the number of risk alleles carried by that individual for genetic variant.

Parameters used to create the PGS (e.g. effect sizes and variant selection) were derived entirely from the discovery cohort.

### 2.8 Elevated LBT

In our basecase analysis, an elevated LBT was defined as a value exceeding the 90th percentile (Table S2). For albumin and PC, however, where lower values indicate higher risk, an ‘elevated’ LBT was defined equivalently as a value below the 10th percentile.

**FIGURE 1:**
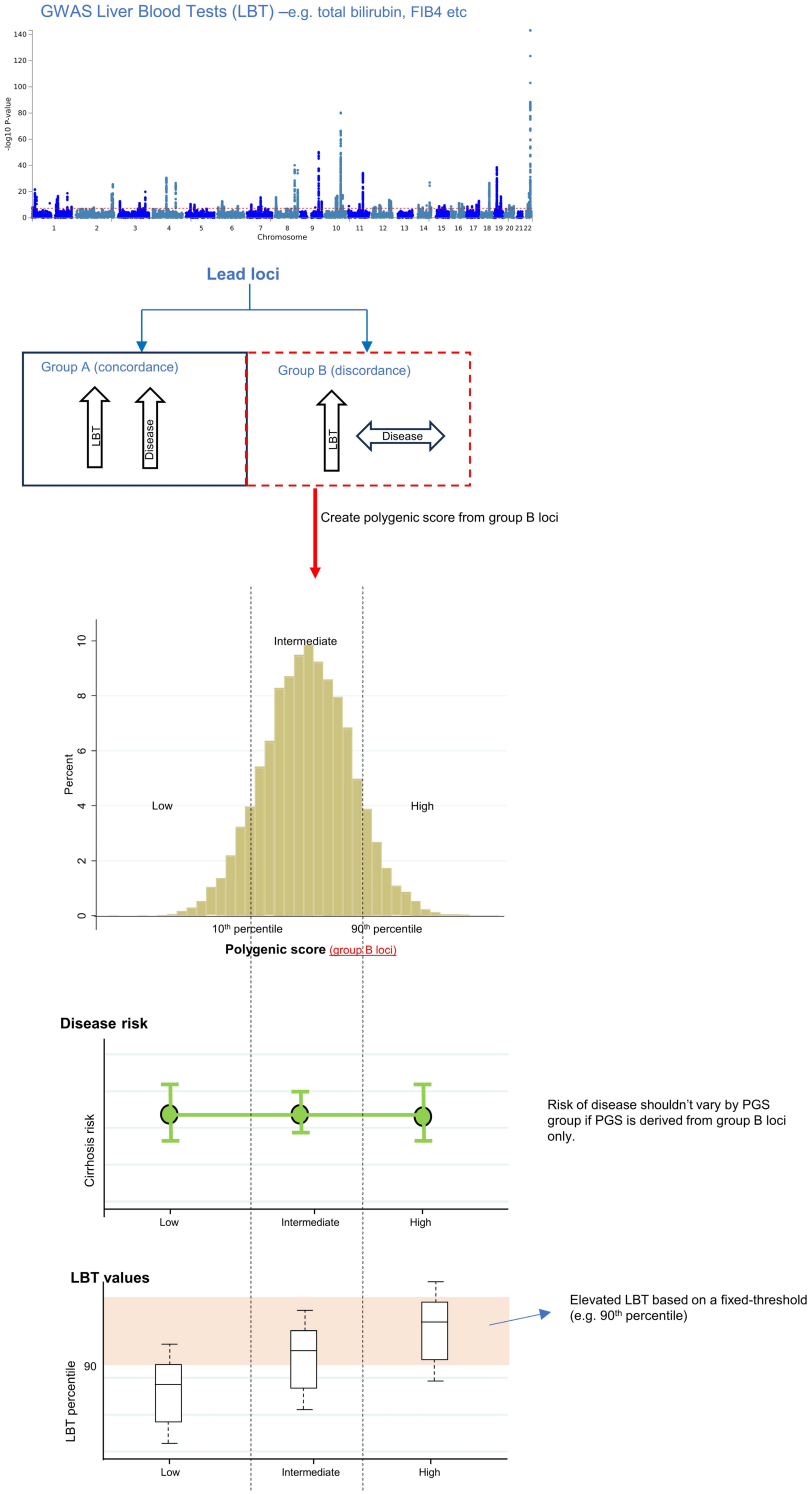
Schematic concept. Key question: can we understand the clinical significance of an ‘elevated LBT’ without knowing an individual's genetic background (i.e. high vs. low PGS).

Figure 1 provides a broad summary of our study hypothesis and methodological approach.

### 2.9 Primary outcome: 10‐year risk of cirrhosis morbidity

Our primary outcome was cirrhosis morbidity presenting as a hospital admission or death related to cirrhosis. International Classification of Diseases (ICD) codes—such as K70.3 (alcohol cirrhosis of liver); K72.1 (chronic hepatic failure) and K76.6 (portal hypertension)—were used to determine whether events were related to cirrhosis. Full details of this codeset are provided in Table S1.

The cumulative incidence of cirrhosis morbidity 10 years after enrolment into UKB was calculated using survival analysis methods. Participants were followed from the date of their UKB interview, through to the earliest of either: (a) the date of a cirrhosis event; (b) date of mortality unrelated to cirrhosis; (c) the study completion date.

The study completion date at the time of analysis was 30 October 2021, 28 February 2018, and 31 July 2021 for participants in England, Wales, and Scotland, respectively.

All estimates of 10‐year risk incorporated death from causes unrelated to cirrhosis as a competing risk event. Ten‐year risk was calculated by fitting Fine‐Gray regression models and then relating the linear predictor to cumulative incidence function via the following equation:

$$\mathrm{CIF}\left(t\right)=1-{\left(1-{\mathrm{CIF}}_{0}\left(t\right)\right)}^{\exp\left(\mathrm{X\beta}\right)}$$

where CIF(*t*) indicates the cumulative incidence function at time *t*; CIF_0_(*t*) is the baseline CIF at time t; and Xβ is the linear predictor of the regression model.^14^

### 2.10 PGS groups

PGS values were computed for individuals in the validation cohort, whose data were not used to develop the PGS. Participants were then categorized into three possible groups based on their PGS value: (a) Low (PGS in decile 1), (b) intermediate (decile 2–9); and (c) high (decile 10).

Each PGS is intended to reflect an individual's natural/baseline LBT level, rather than the risk of disease. The ‘high’ PGS group is analogous to the concept of GS (i.e. elevated LBT without a reciprocal increase in disease risk).

In sensitivity analyses, we also considered a more extreme definition of a high and low PGS, based on a PGS >95th and <5th percentile, respectively.

### 2.11 Added value of PGS

The clinical utility of each PGS was explored by posing the following three questions. Our goal was to determine whether the PGS group enhances the evaluation of the clinical significance of an elevated LBT.

#### 2.11.1 How do LBT values vary by PGS group?

We calculated the proportion of individuals with an elevated LBT value in each PGS group. Moreover, we quantified the association between the PGS (on a continuous scale) and LBT using linear regression. Note that all LBTs were log‐transformed in this latter analysis to achieve approximate normality. Through linear regression, we were also able to derive the R2 value, representing the proportion of variability in the LBT that can be explained by the PGS.

#### 2.11.2 How does 10‐year risk vary by PGS group?

The 10‐year risk of cirrhosis morbidity for individuals in each PGS group was calculated by fitting a Fine‐Gray regression model with PGS group as the only independent variable. We also quantified the association between PGS and cumulative incidence of cirrhosis morbidity with PGS included as a continuous variable.

#### 2.11.3 How does 10‐year risk for individuals with an elevated LBT vary by PGS group?

A Fine‐Gray regression model was fitted, including PGS group and elevated LBT status as independent variables. Crucially, interaction terms between these two variables were also included. This permitted the estimation of 10‐year risk for an elevated LBT, stratified by PGS group. *P*‐values for differences in 10‐year risk were derived from the coefficients of the underlying regression model. Specifically, we added the PGS coefficient to the corresponding interaction coefficient using the lincom command in Stata. Mathematically, the sum of these two coefficients determines the departure in 10‐year risk (if any) from the reference group; the null hypothesis is that the sum of these coefficients is zero.

### 2.12 Sensitivity analysis

#### 2.12.1 Definition of elevated LBT

In sensitivity analyses, we included a more stringent definition of an elevated LBT, based on exceeding the 95th percentile (or equivalently, below the 5th percentile for albumin and platelet count).

We also performed an alternative analysis where the ‘upper limit of normal’ was used to define an elevated LBT. The upper limit of normal was defined as the LBT's 95th percentile among participants with a healthy BMI (≤25 kg/m^2^), without excess alcohol intake (<14 units/weeks), and with no record of prior liver disease. This definition is broadly comparable to a previous study by Valenti et al.^15^

#### 2.12.2 Subgroup analysis

We also calculated 10‐year risk among: (i) the subgroup of participants with risk factors for metabolic dysfunction‐associated steatotic liver disease (MASLD), and (ii) the subgroup at risk of alcohol liver disease (self‐reported intake>14 units/week). Note, individuals with evidence of viral hepatitis, autoimmune disease and genetic liver disease (hemochromatosis or alpha antitrypsin) were excluded from both subgroups. MASLD risk factors were defined as: BMI >30 kg/m^2^, type 2 diabetes, or abdominal obesity (waist: hip ratio >1 if male and >.9 if female).

#### 2.12.3 Outcome event

We repeated our base analysis, but with all‐cause mortality as the outcome, rather than cirrhosis morbidity.

## 3 RESULTS

### 3.1 Study cohort

The discovery sample included *n* = 147 582 to 166 594 individuals, depending on the LBT (see Figure S1). The mean age was 57.8 years and 53.1% were female. The median BMI was 26.7 and the mean alcohol intake was 9.0 units per week (Table S3). Between 763 and 858 cirrhosis events were observed during a mean follow‐up duration of 11.6 years. The 10‐year risk of cirrhosis morbidity was .39% (95% CI: .36–.42).

In the validation cohort, the number of individuals varied from *n* = 156 961 to 171 940, depending on the LBT. The baseline characteristics were similar to the discovery cohort. There were 956 to 1055 cirrhosis events observed during a mean follow‐up duration of 12.7 years (Table S3). The 10‐year risk of cirrhosis morbidity was .41% (95% CI: .38–.44).

The number and overlap of participants included in subgroup analyses are shown in Figure S2.

### 3.2 GWAS analysis and derivation of PGS

6.2 million genetic variants passed our aforementioned QC tests. Genome‐wide association analyses performed on the discovery cohort identified between 91 and 456 lead genomic loci, for each LBT (Table S4, Figures S3–S12). Detailed annotation of the lead loci can be found in Tables S5–S14.

Most lead loci were group B loci, not associated with incident cirrhosis morbidity. Figures S13–S22 provide a graphical representation of each lead variant's association with the LBT and its association with cirrhosis morbidity. The proportion of group B loci varied from a minimum of 136/144 (94%) for ALT to a maximum of 100% (e.g. 184/184 for bilirubin; and 102/102 for albumin) (Table S12).

**FIGURE 2:**
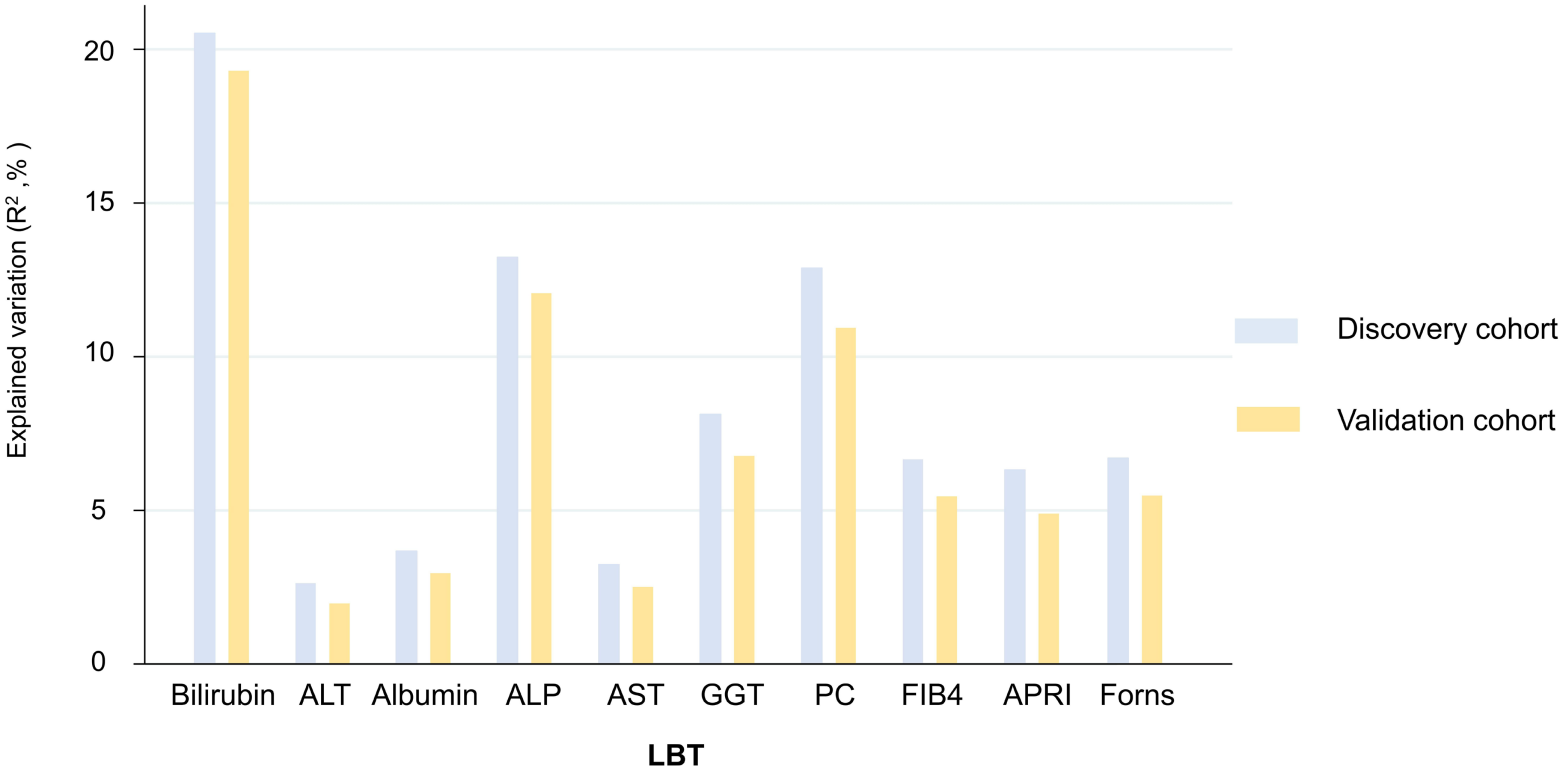
Proportion (%) of variation in liver blood test (LBT) values explained by polygenic score. ALT, alanine aminotransferase; APRI, aspartate aminotransferase ratio; AST, aspartate aminotransferase; FIB4, Fibrosis 4 index; GGT, gamma‐glutamyl transferase; PC, Platelet count.

The amount of variability in LBT values explained by each PGS ranged from a minimum of 2.0% for ALT to a maximum of 19.3% for total bilirubin (Figure 2).

### 3.3 Interpreting elevated LBTs

#### 3.3.1 An elevated LBT is associated with a greater risk of cirrhosis morbidity

**FIGURE 3:**
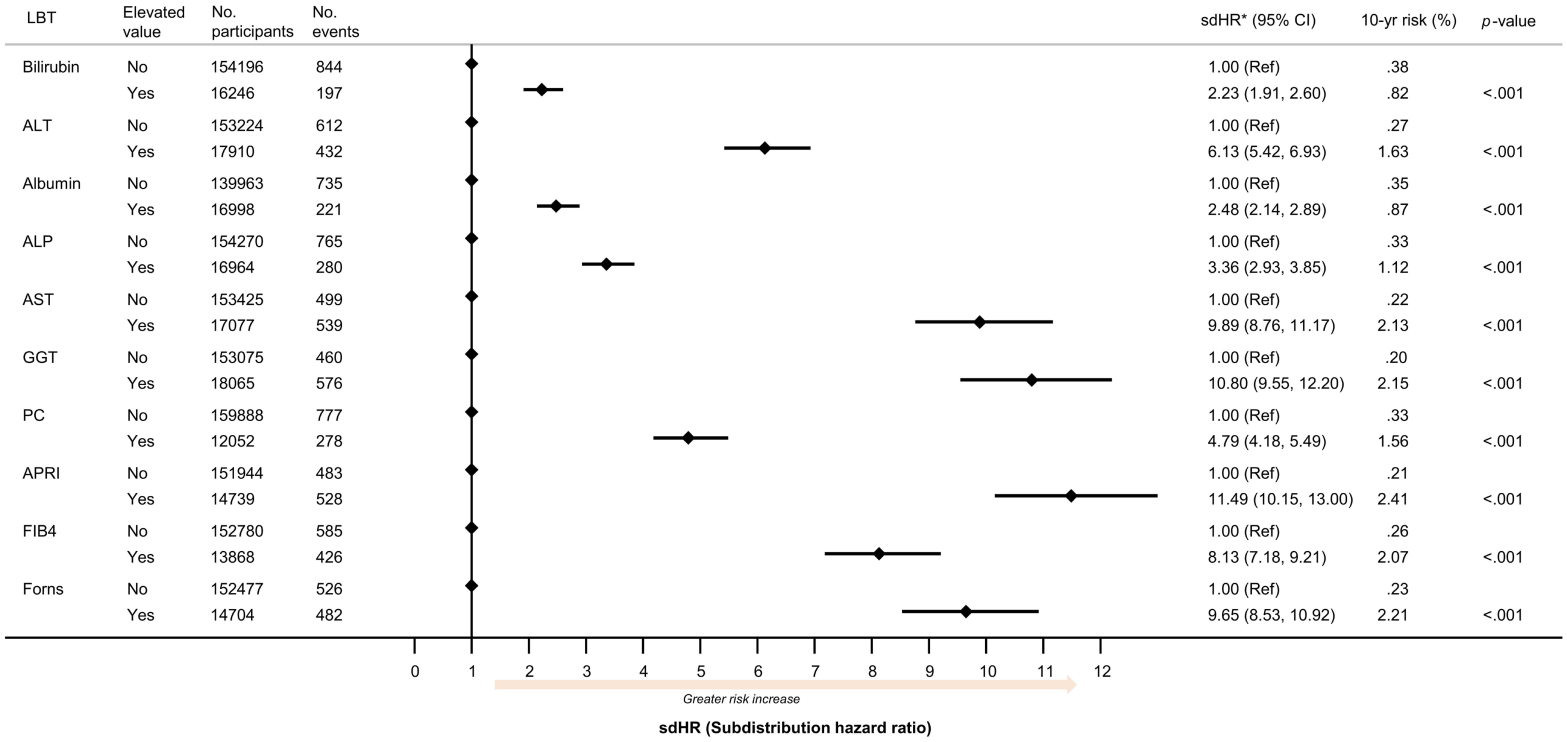
Association between elevated liver blood test (LBT) value and risk of cirrhosis morbidity. Data are derived from participants in the validation subset. Association are expressed in terms of the sdHR. ALT, alanine aminotransferase; APRI, aspartate aminotransferase ratio; AST, aspartate aminotransferase; FIB4, Fibrosis 4 index; GGT, gamma‐glutamyl transferase; PC, Platelet count; sdHR, subdistribution hazard ratio. Elevated LBT = value >90th percentile for Bilirubin, ALT, ALP, AST, GGT, APRI, FIB4. For albumin and PC however, where low values are detrimental, an elevated LBT = value <10th percentile.

Elevated LBTs were associated with a greater risk of cirrhosis morbidity; however, the level of risk elevation was much greater for some LBTs than for others (Figure 3). The risk elevation was lowest for total bilirubin (sdHR: 2.23; 95% CI: 1.91–2.60; *p* < .001) and greatest for APRI (sdHR: 11.49; 95% CI: 10.15–13.00; *p* < .001).

#### 3.3.2 LBT values vary by PGS group

**FIGURE 4:**
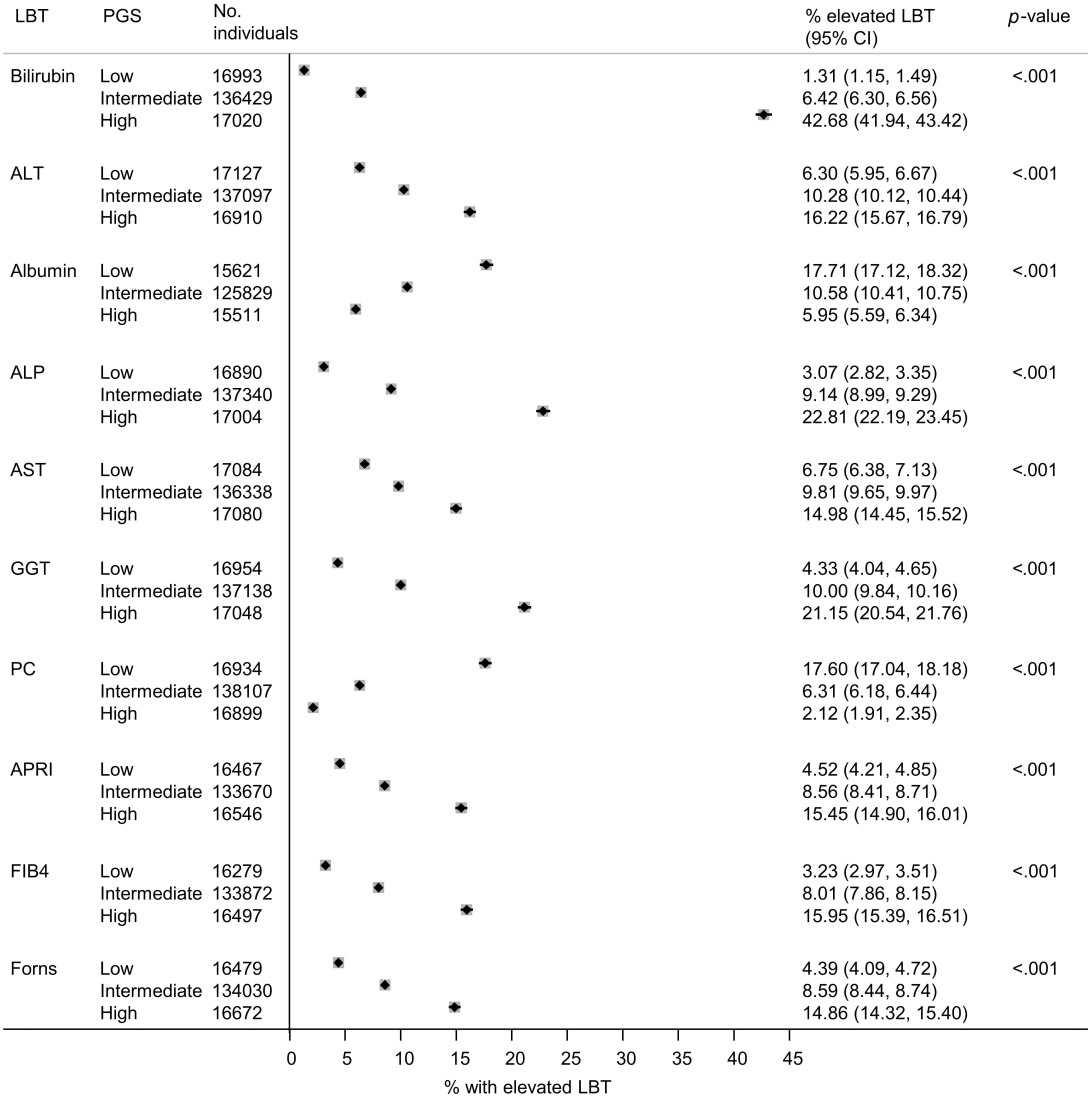
Proportion of individuals with an elevated liver blood test (LBT) value according to polygenic score (PGS). All data shown relate to participants in the validation subset. ALT, alanine aminotransferase; APRI, aspartate aminotransferase ratio; AST, aspartate aminotransferase; FIB4, Fibrosis 4 index; GGT, gamma‐glutamyl transferase; PC, Platelet count. Elevated LBT = value >90th percentile for Bilirubin, ALT, ALP, AST, GGT, APRI, FIB4. For albumin and PC however, where low values are detrimental, an elevated LBT = value <10th percentile. Low PGS = decile 1; intermediate PGS = decile 2–9; high PGS = decile 10.

LBT values were markedly different for people with a low PGS versus a high PGS (Figure 4). This was most apparent for bilirubin, where 42.7% (95% CI: 41.9–43.4) of people with a high PGS had an elevated bilirubin level versus only 1.3% (95% CI: 1.2–1.5) in the low PGS group. However, the same pattern was also apparent for other LBTs. For example, for FIB‐4, 16.0% (95% CI: 15.4–16.5) of people with a high PGS had an elevated FIB‐4 versus only 3.2% (95% CI: 3.0–3.5) with a low PGS. Similarly, for GGT, 21.2% (95% CI:20.5–21.8) of people with a high PGS had an elevated GGT versus only 4.3% (95% CI: 4.0–4.7) in the low PGS group.

#### 3.3.3 PGS does not associate with cirrhosis morbidity risk

**FIGURE 5:**
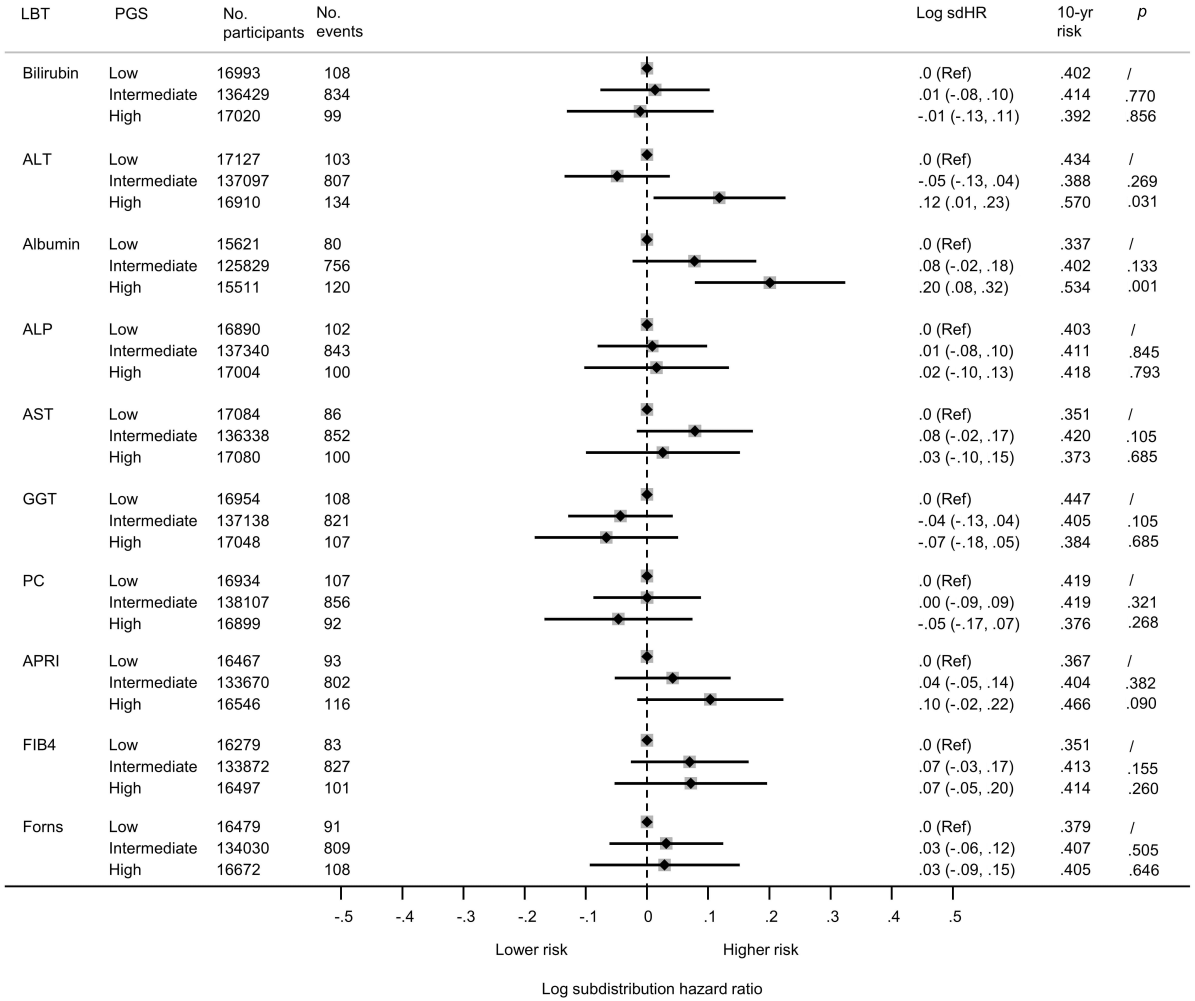
Association between polygenic score (PGS) and cirrhosis morbidity risk. Data are deived from participants in the validation subset. Association are expressed in terms of the sdHR, and pressented on the log scale, which are symmetrical around the point of null effect (i.e. zero). ALT, alanine aminotransferase; APRI, aspartate aminotransferase ratio; AST, aspartate aminotransferase; FIB4, Fibrosis 4 index; GGT, gamma‐glutamyl transferase; PC, Platelet count; sdHR, subdistribution hazard ratio. Low PGS = decile 1; intermediate PGS = decile 2–9; high PGS = decile 10.

Despite the difference in LBT values (Figure 4), the risk of cirrhosis morbidity was generally similar for people with a high PGS and a low PGS (Figure 5).

For example, for GGT, 4.3% of individuals with a low PGS had an elevated GGT value and the 10‐year risk of cirrhosis morbidity was .45%. Conversely, in the high PGS group, although far more had an elevated GGT value (21.2%), the 10‐year risk was no higher than in the low PGS group (.38%). Another example is total bilirubin. In the low PGS group, 1.3% had an elevated bilirubin and the 10‐year risk was .40%. However, in the high PGS group, the proportion with an elevated value was >30 times higher (i.e. 42.7%) yet the 10‐year risk was similar at .39%. This same pattern was apparent for other LBTs, such as FIB‐4 (Figures 4 and 5).

Albumin and ALT were exceptions to this pattern, as for these LBTs, individuals in the ‘high’ PGS group had a significantly higher risk of cirrhosis morbidity (*p* < .01).

#### 3.3.4 The prognosis associated with an elevated LBT varies appreciably by PGS group

**FIGURE 6:**
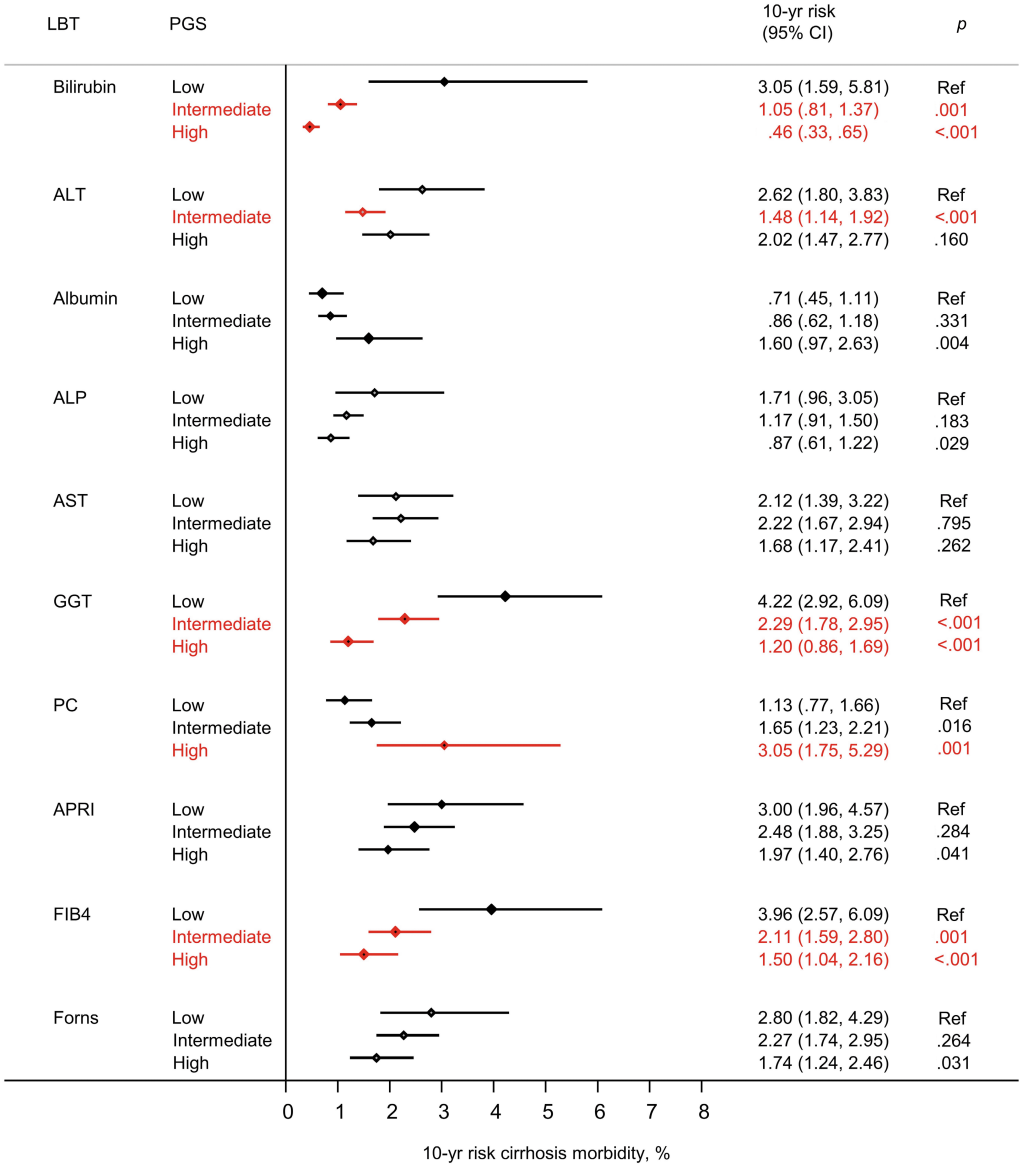
Ten‐year risk of cirrhosis morbidity for participants with an elevated liver blood test (LBT) value, according to polygenic score. Ten years risks significantly different from the references group (at <=.001) are highlighted in red. Estimates of 10 years risk are derived from Fine‐Grayregression models comprising participants in the validation subset. The full regression models are shown in Table S15. ALT, alanine aminotransferase; APRI, aspartate aminotransferase ratio; AST, aspartate aminotransferase; FIB4, Fibrosis 4 index; GGT, gamma‐glutamyl transferase; PC, Platelet count. Low PGS = decile 1; intermediate PGS = decile 2–9; high PGS = decile 10. Elevated LBT = value >90th percentile for Bilirubin, ALT, ALP, AST, GGT, APRI, FIB4. For albumin and PC however, where low values are detrimental, an elevated LBT = value <10th percentile.

The prognosis associated with an elevated LBT (i.e. 10‐year risk) was markedly different for individuals with a low versus high PGS (Figure 6). The differences were most pronounced for bilirubin, where the 10‐year risk was 3.1% (95% CI: 1.6–.5.8) for individuals with an elevated bilirubin in the low PGS group versus .5% (95% CI: .3–.7) in the high PGS group (*p* < .001).

However, significant differences were also apparent in relation to GGT, FIB‐4, and platelet count (Figure 6). For example, for GGT, the 10‐year risk was 4.2% (95% CI: 2.9–.6.1) for individuals with an elevated GGT in the low PGS group versus 1.2% (95% CI: .9–1.7) in the high PGS group (*p* < .001). Another example relates to FIB‐4, where the 10‐year risk was 4.0% (95% CI: 2.6–.6.1) for individuals with an elevated FIB‐4 in the low PGS group versus 1.5% (95% CI: 1.0–2.2) in high PGS group (*p* < .001).

The regression modelling parameters on which all PGS‐stratified 10‐year risk estimates are based are provided in Table S15.

### 3.4 Sensitivity and post hoc analyses

In sensitivity analyses, differences in 10‐year risk by PGS group were most consistent for bilirubin, platelet count, GGT, and FIB‐4 (Table 1).

**TABLE 1:** Ten‐year risk of cirrhosis morbidity for participants with an elevated liver blood test (LBT), by polygenic score: basecase and sensitivity analyses (SA).

LBT	PGS	Basecase analysis (elevated LBT= >90th percentile)					SA1: elevated LBT= >95th percentile					SA2: elevated LBT = upper limit of normal					SA3: subgroup with MAFLD risk factors					SA4: subgroup at risk of alcohol liver disease					SA5: as per basecase, except outcome = all cause mortality				
		Definition of elevated LBT	10‐year risk	Lower 95% CI	Upper 95% CI	*p*‐value	Definition of elevated LBT	10‐year risk	Lower 95% CI	Upper 95% CI	*p*‐value	Definition of elevated LBT	10‐year risk	Lower 95% CI	Upper 95% CI	*p*‐value	Definition of elevated LBT	10‐year risk	Lower 95% CI	Upper 95% CI	*p*‐value	Definition of elevated LBT	10‐year risk	Lower 95% CI	Upper 95% CI	*p*‐value	Definition of elevated LBT	10‐year risk	Lower 95% CI	Upper 95% CI	*p*‐value
Bilirubin	Low	>13.9 umol/L	3.05	1.59	5.81	Ref	>17.3 umol/L	4.78	1.51	14.64	Ref	>16.3 umol/L	4.04	1.49	10.75	Ref	>13.9 umol/L	6.03	2.46	14.40	Ref	>13.9 umol/L	4.01	1.82	8.73	Ref	>17.3 umol/L	13.3	8.9	19.5	Ref
	Int		1.05	.81	1.37	.001		1.41	1.04	1.90	.040		1.34	1.01	1.78	.031		1.95	1.36	2.78	.013		1.29	.89	1.88	.004		10.7	9.8	11.6	.284
	High		.46	.33	.65	<.001		.45	.30	.67	<.001		.49	.33	.71	<.001		.87	.54	1.40	<.001		.53	.31	.89	<.001		9.2	8.4	10.2	.077
ALT	Low	>37.8 U/L	2.62	1.80	3.83	Ref	>47.0 U/L	4.17	2.77	6.24	Ref	>30.9 U/L	1.88	1.30	2.72	Ref	>37.8 U/L	2.56	1.54	4.25	Ref	>37.8 U/L	2.66	1.51	4.66	Ref	>47.0 U/L	11.8	9.7	14.3	Ref
	Int		1.48	1.14	1.92	<.001		2.25	1.75	2.90	.001		1.04	.78	1.38	<.001		1.86	1.32	2.60	.139		1.62	1.10	2.41	.036		10.5	9.7	11.3	.246
	High		2.02	1.47	2.77	.160		2.48	1.77	3.48	.019		1.49	1.07	2.07	.149		2.37	1.56	3.59	.756		2.41	1.51	3.83	.71		11.9	10.5	13.5	.919
Albumin	Low	<42.0 g/L	.71	.45	1.11	Ref	<41.0 g/L	.89	.53	1.48	Ref	<41.2 g/L	.83	.50	1.38	Ref	<42.0 g/L	1.21	.67	2.19	Ref	<42.0 g/L	1.26	.66	2.40	Ref	<41.0 g/L	14.9	13.2	16.8	Ref
	Int		.86	.62	1.18	.331		1.21	.89	1.66	.203		1.15	.85	1.57	.169		1.18	.77	1.82	.914		1.24	.78	1.98	.961		16.3	15.2	17.6	.104
	High		1.60	.97	2.66	.004		1.87	.99	3.54	.047		1.62	.85	3.07	.075		2.23	1.13	4.37	.099		1.38	.53	3.57	.860		18.1	15.2	21.3	.045
ALP	Low	>113 U/L	1.71	.96	3.05	Ref	>125.5 U/L	2.73	1.42	5.21	Ref	>121.9 U/L	2.46	1.32	4.58	Ref	>113 U/L	2.08	.94	4.57	Ref	>113 U/L	2.52	.91	6.86	Ref	>125.5 U/L	19.5	15.8	23.9	Ref
	Int		1.17	.91	1.50	.183		1.75	1.35	2.25	.170		1.59	1.24	2.04	.158		1.53	1.09	2.14	.428		1.64	1.14	2.36	.403		16.3	15.3	17.5	.097
	High		.87	.61	1.22	.029		1.22	.84	1.78	.023		1.15	.80	1.65	.024		1.32	.83	2.08	.275		1.11	.65	1.91	.138		14.3	12.9	15.8	.006
AST	Low	>34.7 U/L	2.12	1.39	3.22	Ref	>40.1 U/L	3.38	2.16	5.28	Ref	>34.1 U/L	2.01	1.32	3.06	Ref	>34.7 U/L	2.47	1.39	4.36	Ref	>34.7 U/L	2.61	1.47	4.63	Ref	>40.1 U/L	16.4	14.0	19.2	Ref
	Int		2.22	1.67	2.94	.795		3.60	2.76	4.69	.747		2.13	1.60	2.82	.747		2.95	2.02	4.30	.447		2.58	1.71	3.87	.95		13.7	12.8	14.7	.031
	High		1.68	1.17	2.41	.262		2.97	2.08	4.23	.574		1.54	1.07	2.21	.190		2.62	1.64	4.18	.828		1.74	1.03	2.96	.155		12.5	10.9	14.2	.006
GGT	Low	>67.0 U/L	4.22	2.92	6.09	Ref	>94.7 U/L	5.88	3.94	8.73	Ref	>53.2 U/L	2.84	1.97	4.10	Ref	>67.0 U/L	5.53	3.38	8.98	Ref	>67.0 U/L	4.43	2.52	7.70	Ref	>94.7 U/L	20.0	16.8	23.8	Ref
	Int		2.29	1.78	2.95	<.001		3.68	2.91	4.66	.009		1.65	1.28	2.14	<.001		2.95	2.02	4.29	.001		2.22	1.40	3.52	<.001		15.7	14.6	16.8	.006
	High		1.20	.86	1.69	<.001		1.96	1.40	2.74	<.001		.96	.69	1.35	<.001		1.50	.92	2.46	<.001		1.31	.75	2.29	<.001		13.7	12.3	15.3	<.001
PC	Low	<180 10^9^ cells/L	1.13	.77	1.66	Ref	<167.0109 cells/L	1.59	1.08	2.34	Ref	<174.5 10^9^ cells/L	1.27	.86	1.86	Ref	<180 10^9^ cells/L	2.77	1.69	4.52	Ref	<180 10^9^ cells/L	1.05	.59	1.87	Ref	<167.0 10^9^ cells/L	12.3	10.9	13.9	Ref
	Int		1.65	1.23	2.21	.016		2.19	1.64	2.92	.064		1.88	1.41	2.51	.017		2.74	1.81	4.14	.967		2.11	1.41	3.17	.005		14.9	13.7	16.1	.002
	High		3.05	1.75	5.29	.001		3.28	1.68	6.33	.040		3.62	2.06	6.33	<.001		3.00	1.24	7.15	.856		3.23	1.35	7.60	.016		15.8	11.9	20.7	.097
APRI	Low	>.41	3.00	1.96	4.57	Ref	>.49	5.48	3.59	8.33	Ref	>.40	2.81	1.84	4.28	Ref	>.41	3.63	2.01	6.50	Ref	>.41	3.71	2.04	6.72	Ref	>.49	16.6	13.6	20.0	Ref
	Int		2.48	1.88	3.25	.284		4.05	3.08	5.32	.097		2.32	1.76	3.06	.278		3.46	2.34	5.11	.847		2.71	1.77	4.14	.185		14.9	13.9	16.0	.295
	High		1.97	1.40	2.76	.041		3.11	2.18	4.43	.008		1.84	1.31	2.59	.038		3.45	2.18	5.46	.854		2.05	1.22	3.44	.035		14.0	12.4	15.8	.130
FIB4	Low	>1.98	3.96	2.57	6.09	Ref	>2.29	7.04	4.44	11.07	Ref	>2.19	6.02	3.85	9.34	Ref	>1.98	7.95	4.59	13.58	Ref	>1.98	6.08	3.37	10.84	Ref	>2.29	20.4	16.7	24.8	Ref
	Int		2.11	1.59	2.80	.001		3.52	2.68	4.62	.001		2.96	2.25	3.90	<.001		3.97	2.73	5.76	.003		2.79	1.88	4.13	.002		20.0	18.7	21.4	.827
	High		1.50	1.04	2.16	<.001		2.27	1.55	3.32	<.001		1.97	1.35	2.87	<.001		4.07	2.59	6.37	.011		1.35	.77	2.37	<.001		18.6	16.7	20.7	.391
Forns	Low	>5.9	2.80	1.82	4.29	Ref	>6.4	3.94	2.43	6.34	Ref	>5.5	2.18	1.45	3.28	Ref	>5.9	3.63	2.06	6.34	Ref	>5.9	2.42	1.25	4.64	Ref	>6.4	24.5	21.0	28.5	Ref
	Int		2.27	1.74	2.95	.264		3.55	2.75	4.56	.638		1.69	1.28	2.24	.127		2.85	1.93	4.22	.288		2.43	1.68	3.52	.980		24.1	22.7	25.7	.855
	High		1.74	1.24	2.46	.031		2.42	1.68	3.46	.059		1.21	.84	1.72	.003		2.85	1.78	4.54	.354		1.94	1.20	3.13	.512		20.0	18.1	22.2	.022

*Note:* Ten year risk significantly different from the reference group (at *p* ≤ 0.001) are highlighted in gray.

Abbreviations: ALT, alanine aminotransferase; APRI, aspartate aminotransferase ratio; AST, aspartate aminotransferase; FIB4, Fibrosis 4 index; GGT, gamma‐glutamyl transferase; PC, Platelet count.

Recent guidelines recommend that people with risk factors for MASLD should be grouped into three FIB‐4 risk strata: <1.3; 1.3–2.67 and >2.67.^16^ In a post hoc analysis, we calculated 10‐year risk by PGS within these FIB‐4 categories. This analysis indicated that for people with a FIB‐4 > 2.67, 10‐year risk varies appreciably by PGS. Variability within the <1.3 and 1.3–2.67 groupings was modest, however (Table S16).

## 4 DISCUSSION

The targeting of clinical resources towards people with elevated biomarker(s) is commonplace in medicine. This applies particularly to Hepatology, where pathways that identify (and trigger further tests and treatments in) individuals with elevated LBTs are widespread.^16^, ^17^, ^18^, ^19^, ^20^, ^21^ However, our study suggests the clinical significance of an elevated LBT varies considerably depending on an individual's genetics, particularly for bilirubin, GGT, FIB‐4, and platelet count. An elevated FIB‐4, for example, could mean either a concerning 10‐year risk of cirrhosis morbidity of 4.0%, or a more modest 10‐year risk of 1.5%. Similarly, an elevated GGT could mean a 10‐year risk of 4.2% or 1.2%. Our analysis suggests this is because LBTs are associated with a multitude of genetic loci, of which the majority do not appear to alter the phenotype of interest (i.e. in this case, the risk of cirrhosis). One example is the rs2361502:T allele in *MROH2A*, which is associated with a substantial increase in total bilirubin levels (*β*: .088), but has no obvious effect on cirrhosis risk (HR:1.02). In other cases, the effect a genetic locus exerts on an LBT can even be athwart to its effect on cirrhosis risk. For example, the well‐known rs28929474:T allele in *SERPINA1* is associated with higher albumin levels (implying lower disease risk), but at the same time, it is strongly associated with a higher risk of cirrhosis. Similarly, the rs429358:C allele in *APOE* is associated with higher FIB‐4 values (implying a higher risk of cirrhosis) but is associated with a lower risk of cirrhosis. In aggregate, these discordances can lead to a sizeable disconnect between an individual's LBT value and their actual risk of disease. This disconnect will be greatest for people in the low and high PGS groups, as defined in our study. Thus, any fixed threshold definition of LBT elevation will always represent—in real terms—a much higher bar for individuals in the low PGS group to surpass versus individuals in the high PGS group. Clinical pathways predicated on elevated LBTs will be at their most suboptimal for people in these low and high PGS groups. The clinical implications of this on an individual basis could be modest but at a population level has the potential to be very significant. A small change in false positive tests will lead to less resources spent on onward investigations which are costly for the health service and time‐consuming and create anxiety for patients. Conversely, an improvement in false negative tests will reduce the burden of those presenting with decompensated liver disease in the future. A more nuanced approach to LBT, which presently is the most common way of diagnosing asymptomatic liver disease, provides a mechanism to impact population liver health.

Our findings beg the question as to how the genetic discordances highlighted might be removed or adjusted for to improve risk stratification pathways. There are two main possibilities. First, personalizing the definition of an ‘elevated’ LBT by applying a higher threshold for people in the ‘high’ PGS group versus the ‘low’ PGS group. Alternatively, one could develop a genetically‐corrected LBT value to allow like‐for‐like comparisons between individuals with different genetic profiles. This was explored in relation to prostate‐specific antigen (PSA) for screening of prostate cancer, where a PGS explained 9.6% of constitutive PSA variation, and using a PGS‐adjusted PSA would avoid up to 31% of negative prostate biopsies.^2^ Our findings suggest there may be merit in applying a similar correction to bilirubin, FIB‐4, GGT, and platelet count.

This study adds to the debate about the value of using germline genetic data for risk stratification. Previous GWAS have identified loci that are directly (causally) associated with liver disease phenotypes such as cirrhosis and HCC.^22^, ^23^, ^24^, ^25^ We and others have shown that these genetic variants add minimal new prognostic information over and above what can already be captured through simple biomarkers.^26^, ^27^ On this basis, it is tempting to conclude that the clinical utility of genetic data for risk prediction is fundamentally limited. However, the present study argues against this, suggesting instead a broader lens must be applied to fully leverage the prognostic potential of genetic data—that is, looking beyond just those variants that are directly associated with disease.

This is the first study to systematically explore natural genetic variability in LBT values from a risk prediction/stratification perspective. Although GS is well known,^4^ we show that this concept applies as much to individuals with genetically low bilirubin levels as genetically high levels. Moreover, we demonstrate that the principles apply to other LBTs beyond bilirubin, particularly FIB‐4, GGT, and platelet count. However, our study has several important limitations that warrant discussion. First, the separation of ‘group A’ versus ‘group B’ loci was carried out on the basis of each loci's association with cirrhosis morbidity in our discovery cohort. This approach is likely to suffer from a high type 2 error rate, particularly for loci with lower allele frequency. In other words, failing to detect associations due to inadequate study power rather than the genuine absence of an association. There is evidence of this particularly in relation to the ALT phenotype where known cirrhosis risk loci were designated as group B (e.g. rs58542926 in *TM6SF2*; see Table S4). However, we do not believe this limitation undermines our conclusions. Indeed, if we were able to distinguish between group A and B loci with greater accuracy, we would expect the difference in 10‐year risk between high and low PGS groups to be even further apart; thus, our conclusions hold *despite* this limitation, not because of it. Nevertheless, fresh perspectives on how to refine this step in our analytical pipeline are welcome. One possibility we considered was to distinguish group A from group B variants using genome annotation informed by multiomics—in effect, to understand the biology of each locus in terms of its downstream effects on CLD (or lack thereof). Resources such as gene ontology, for example, could help to support this.^28^ However, interpreting the downstream biological effects of loci is highly complex and not clear‐cut. Moreover, such information will inevitably be incomplete for many loci; hence, why we decided against this approach.

Second, our study adopted a prognostic focus with hard clinical outcomes—that is, 10‐year risk of cirrhosis morbidity. To understand diagnostic accuracy for pathological aspects of fibrosis, steatosis and inflammation, a different approach would be required. Another caveat is that our findings may not be generalizable to liver disease patients in secondary care. Relatedly, the UKB cohort is not representative of a primary care population nor the UK general population; thus, the incidence rates/10‐year risks reported may not be generalizable to either of these settings. UKB participants are more likely to be female, older, and live in less socioeconomically deprived areas than non‐participants.^29^ However, we do not think that the variation in 10‐year risk by PGS is likely to be affected by this selection bias. Our study does not feature any external validation because we were unable to find a suitable cohort where this could be carried out. Further investigation is needed to corroborate these findings in other cohorts. Another point to note is that our primary endpoint was cirrhosis morbidity rather than cirrhosis per se. Instances of compensated cirrhosis are likely underrepresented by this definition, which we concede is a limitation. On the other hand, it could be argued that a focus on overt disease (i.e. morbidity stemming from cirrhosis) is more relevant from a clinical, patient and population health perspective.

All in all, despite these limitations, our results clearly show that variability in LBTs is partly driven by genetic polymorphisms that have a neutral effect on disease risk. These findings have implications for interpreting high/low test values in clinical practice and suggest an important potential role for genetics in the future to enhance diagnostic precision and contribute to more personalized patient care.

## AUTHOR CONTRIBUTIONS

Author‐specific contributions are outlined in the table below. Hamish Innes acts as the guarantor of the article. All authors have approved the final version of the manuscript.

## FUNDING INFORMATION

Funding for the study was received by UKRI (Project Reference: MR/W014491/1) and National Institute for Health Research Nottingham Digestive Diseases Biomedical Research Centre based at Nottingham University Hospitals NHS Trust and University of Nottingham. This work was also supported by a viral hepatitis fellowship to HI from the Medical Research Foundation (Reference: C0825). The funders had no involvement in study design, writing the report or decision for publication.

## CONFLICT OF INTEREST STATEMENT

There are no conflicts of interest to disclose.

## Supporting information


Figure S1:
Figure S2:
Figure S3:
Figure S4:
Figure S5:
Figure S6:
Figure S7:
Figure S8:
Figure S9:
Figure S10:
Figure S11:
Figure S12:
Figure S13:
Figure S14:
Figure S15:
Figure S16:
Figure S17:
Figure S18:
Figure S19:
Figure S20:
Figure S21:
Figure S22:

Table S1:

## Data Availability

This research has been conducted using the United Kingdom (UK) Biobank Resource (application number: 8764). Data from the UK Biobank are accessible to all bonafide researchers. For information about the application procedure, please see: https://www.ukbiobank.ac.uk/enable‐your‐research/apply‐for‐access.
