# Supplementary material for: Interpreting elevated liver blood test results through a genetic lens: A genome‐wide association study
Source: Liver Int. 2024 Oct 19;44(12):3260–73. doi: 10.1111/liv.16114 (PMC11586890; doi:10.1111/liv.16114)

**Supplementary Figures.**

**Interpreting elevated liver blood test results through a genetic lens: a genome-wide association study**

Hamish Innes, Stephan Buch, Timothy J Kendall, Jonathan A Fallowfield, Indra Neil Guha


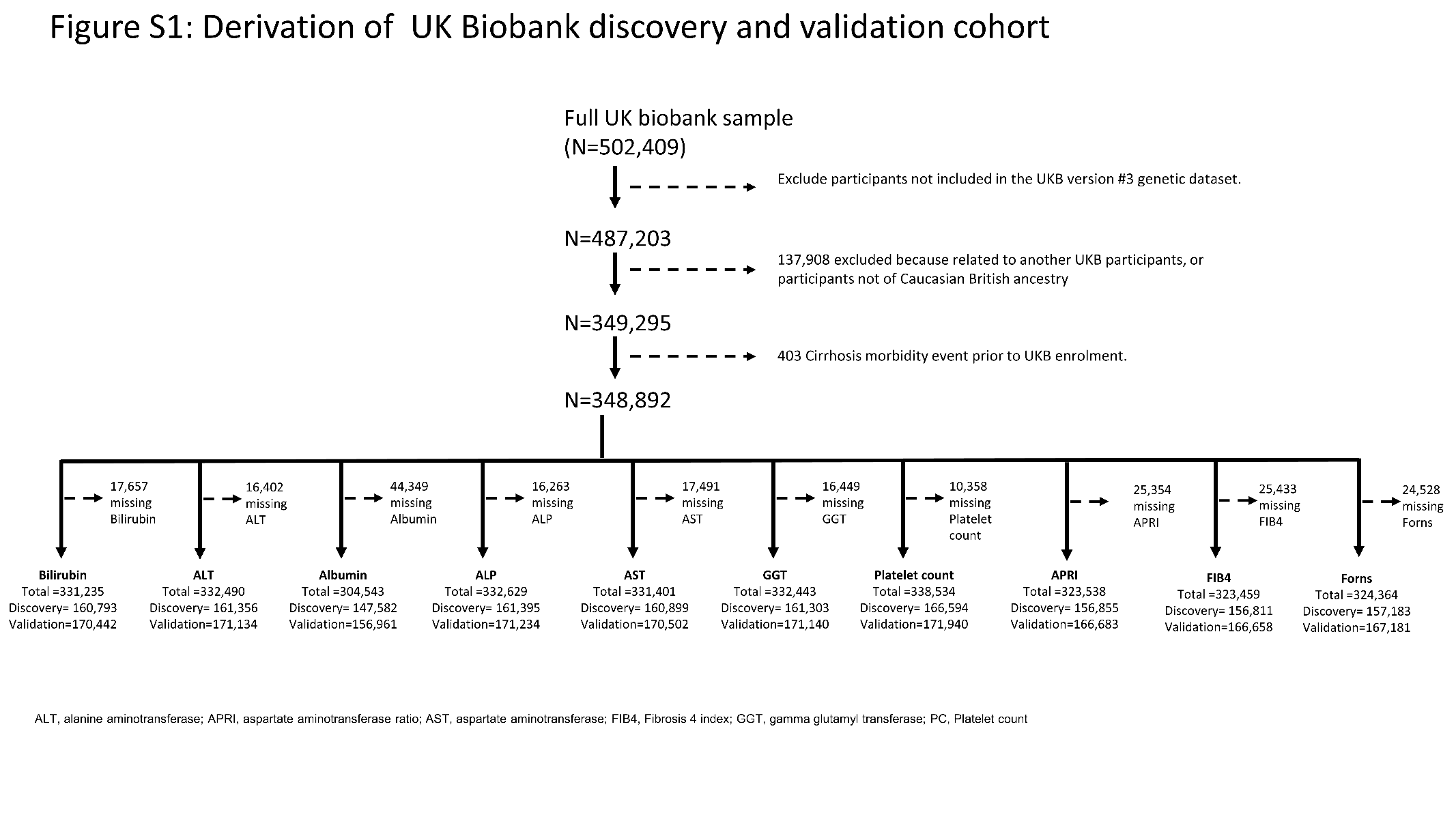


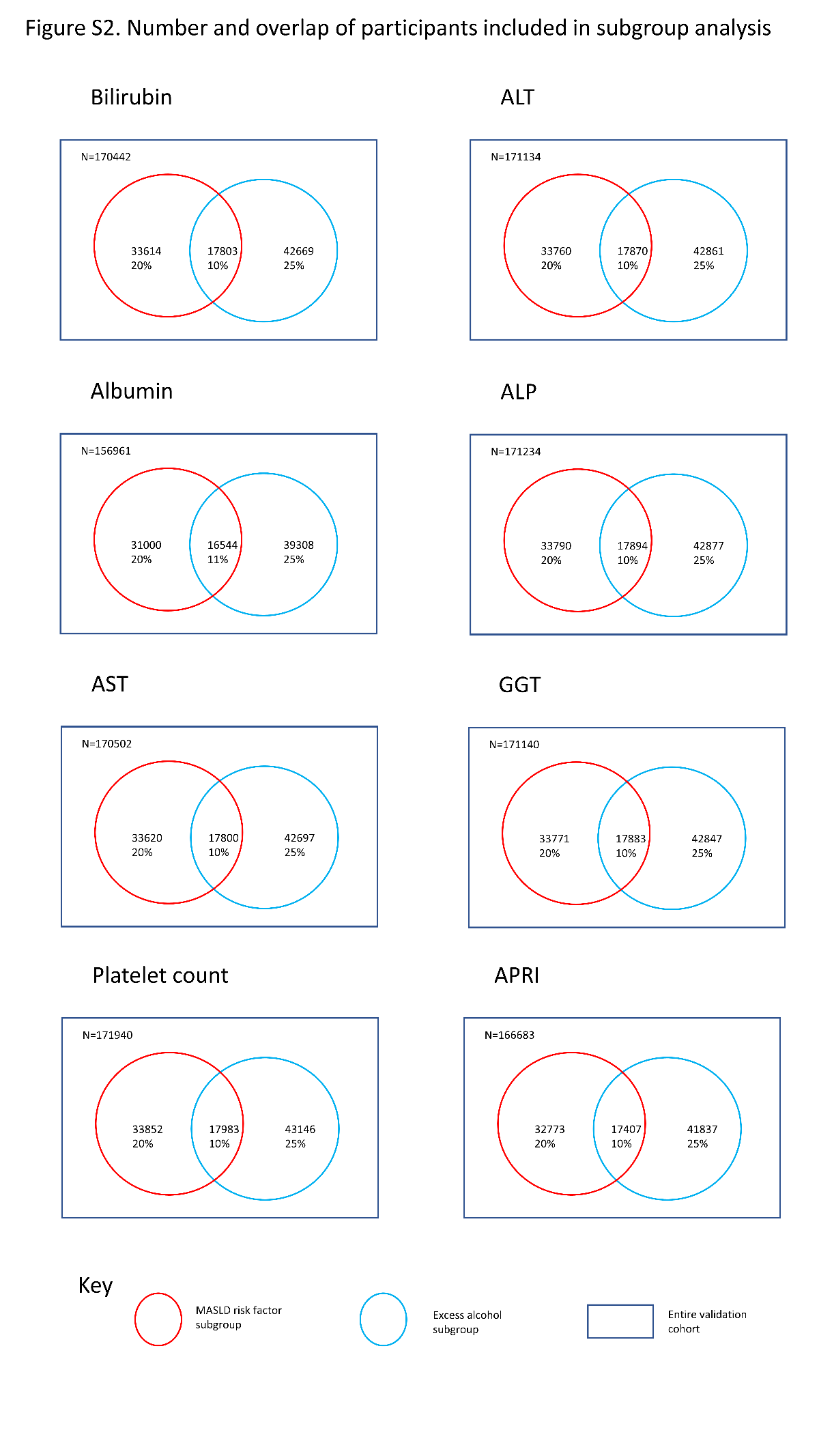


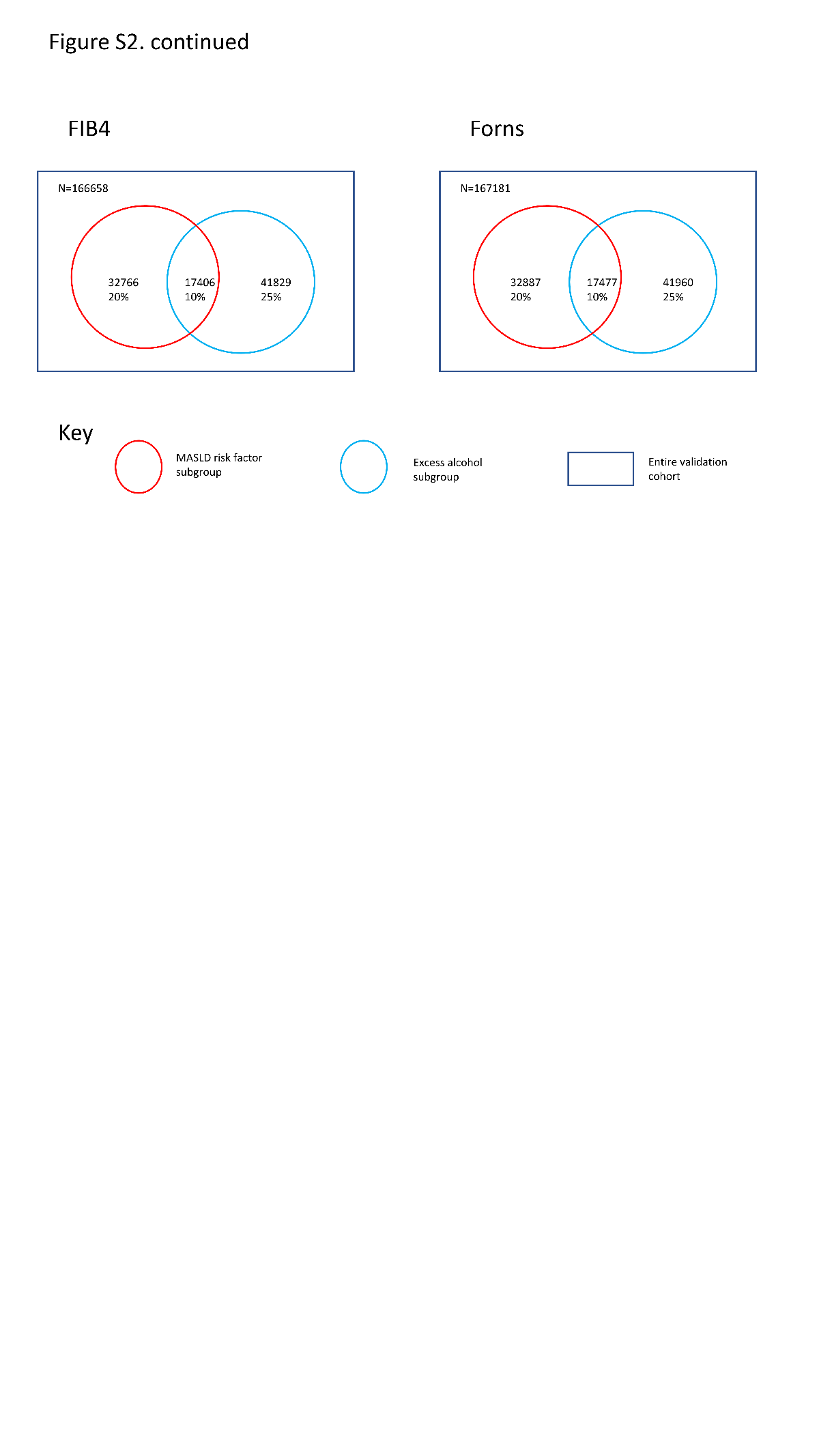


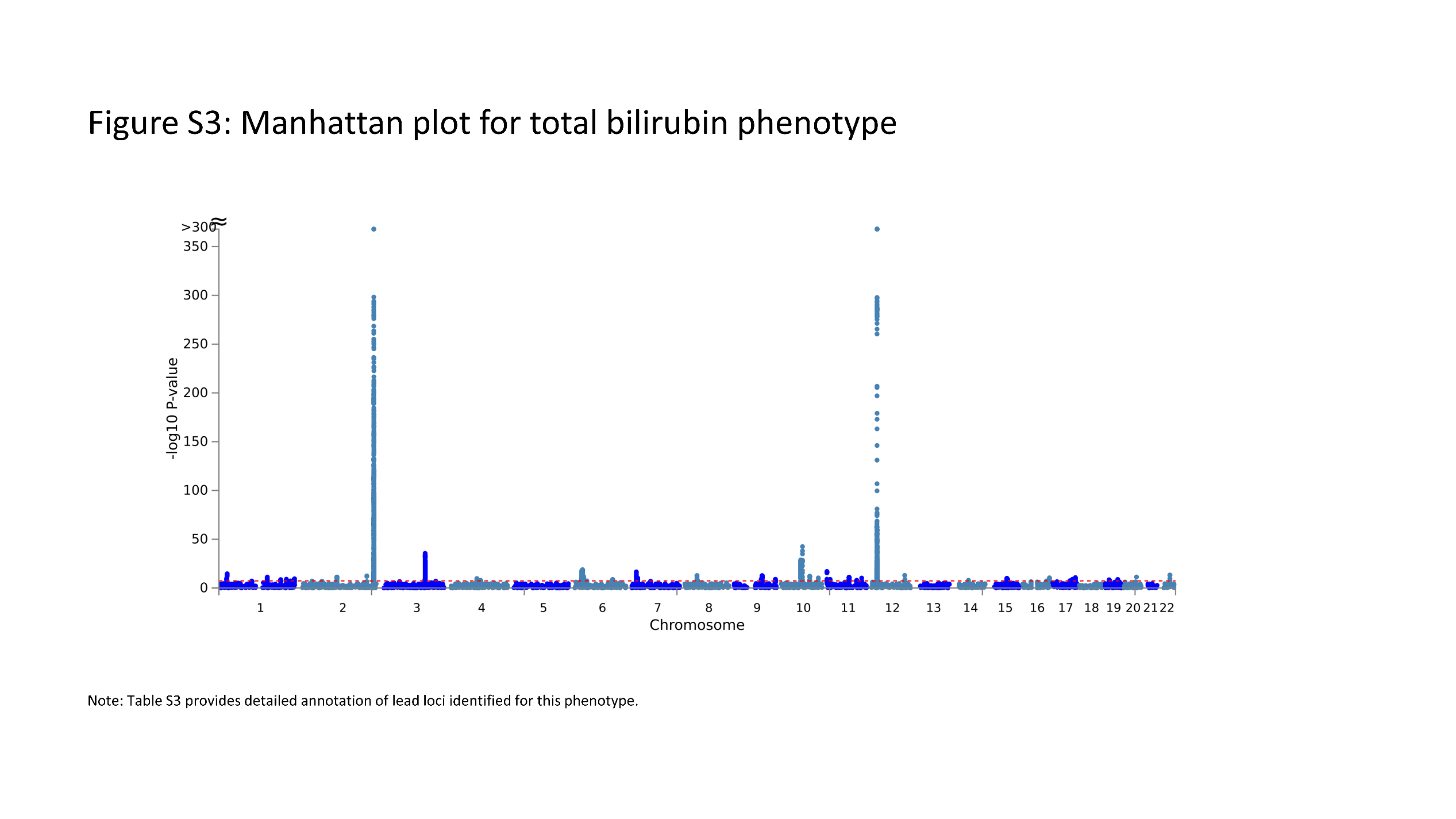


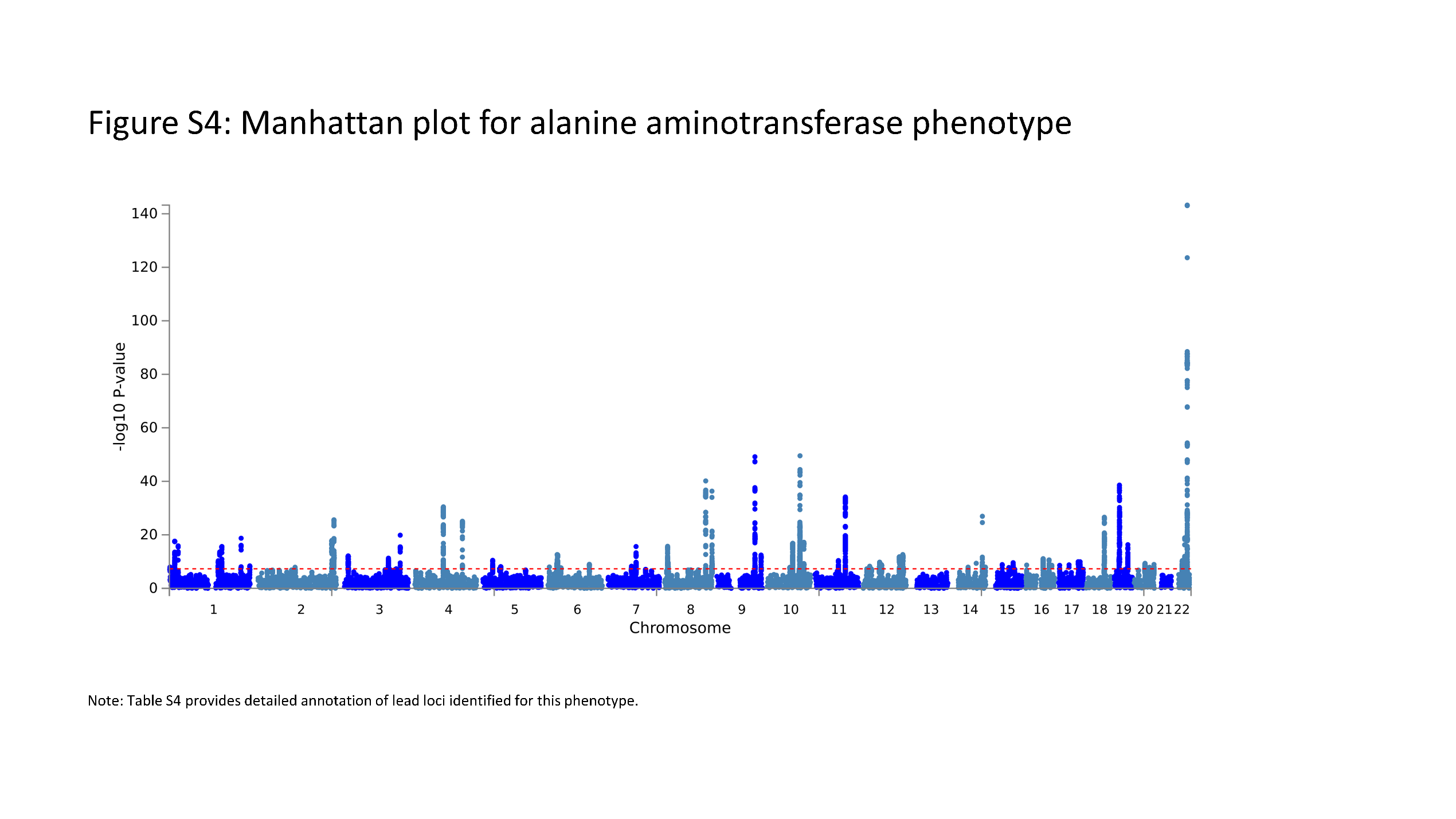


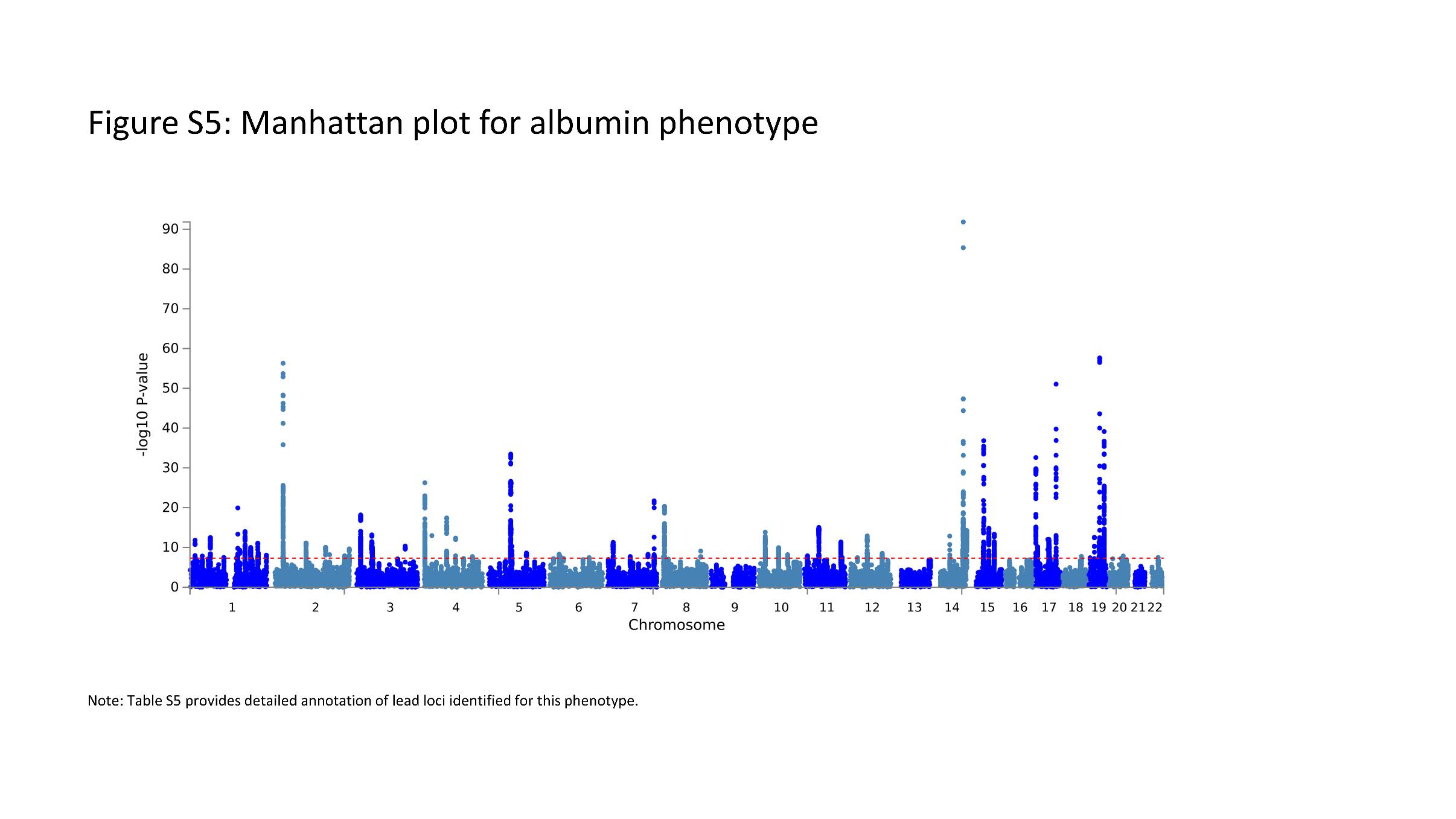


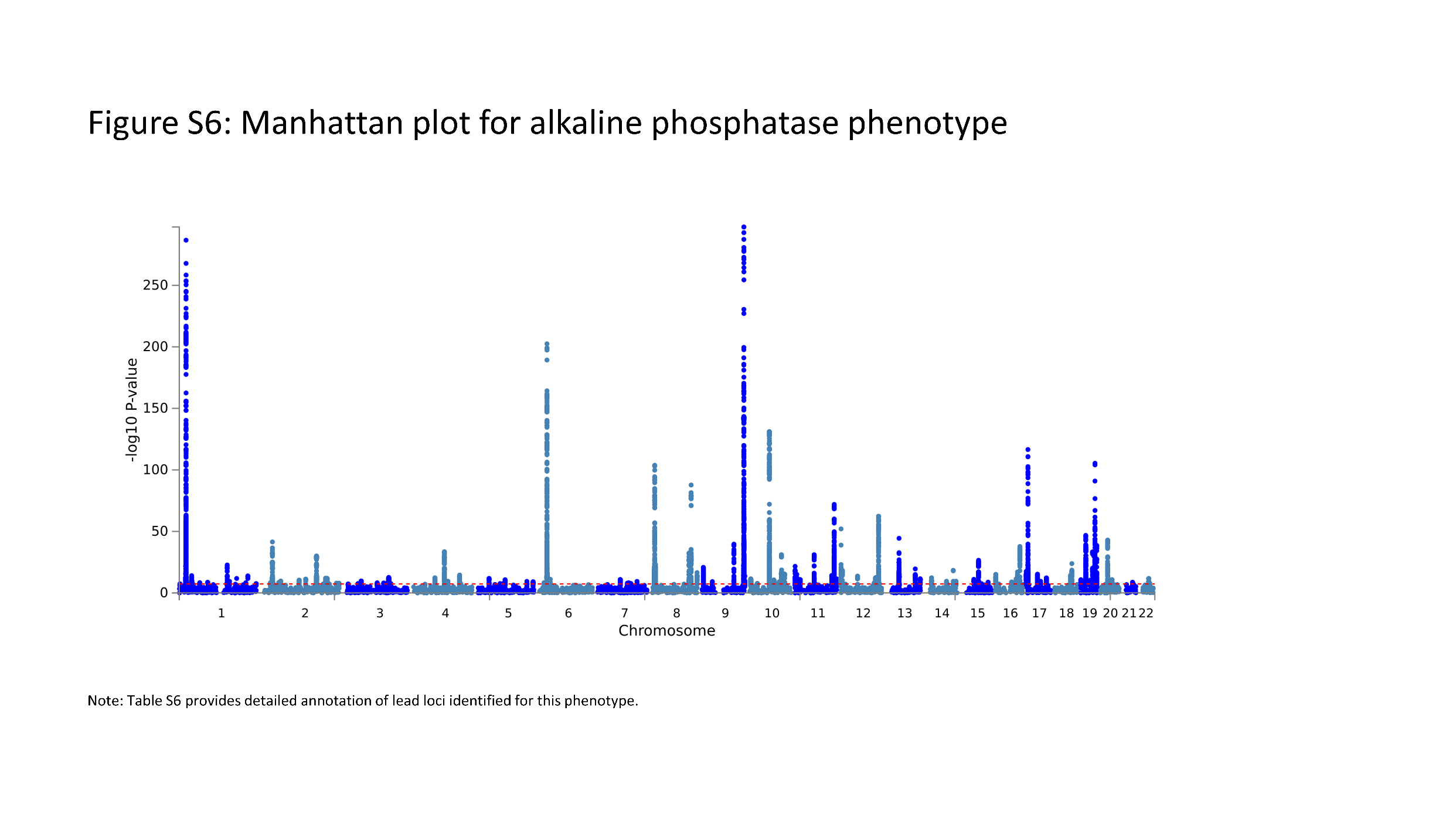


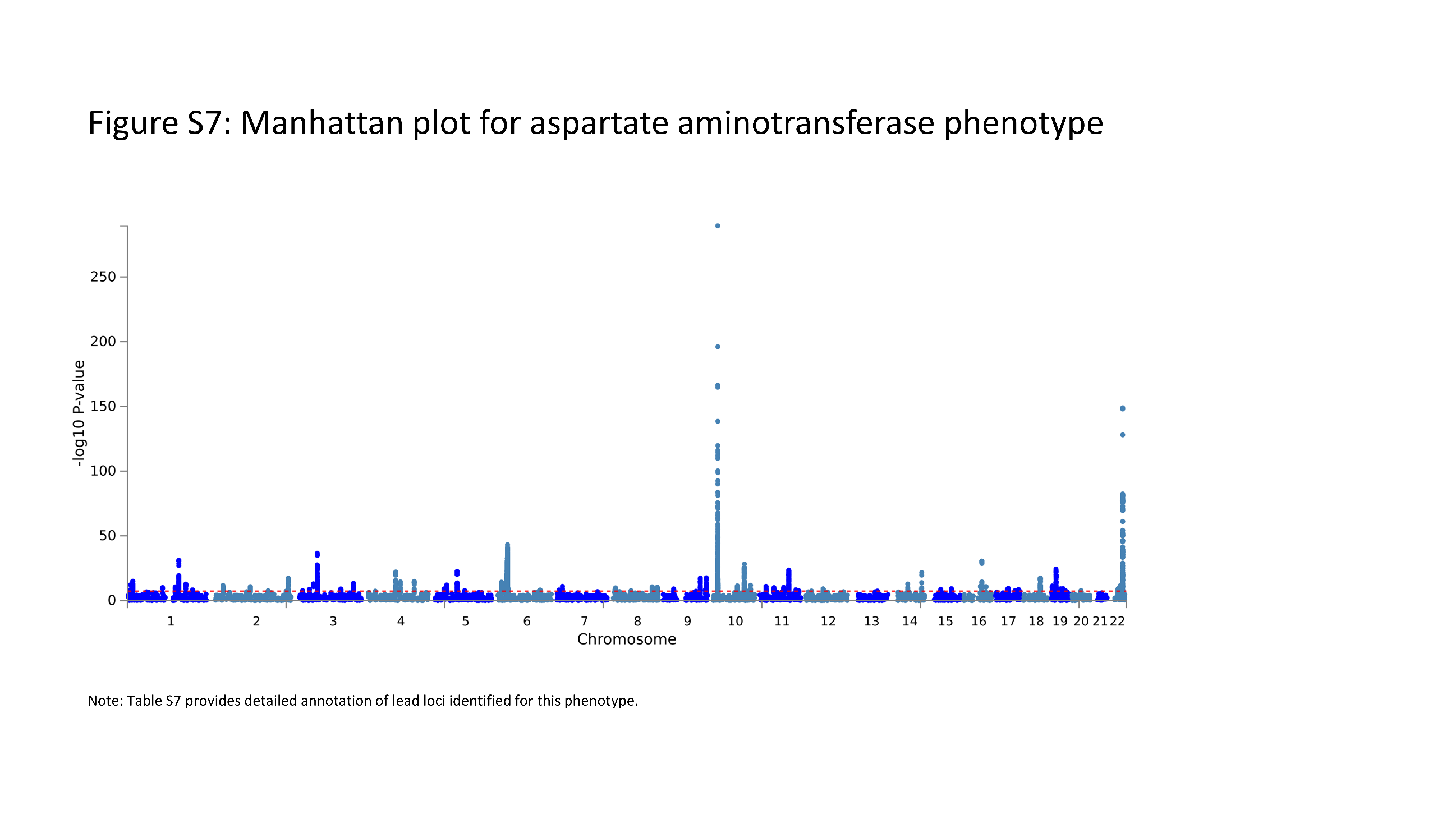


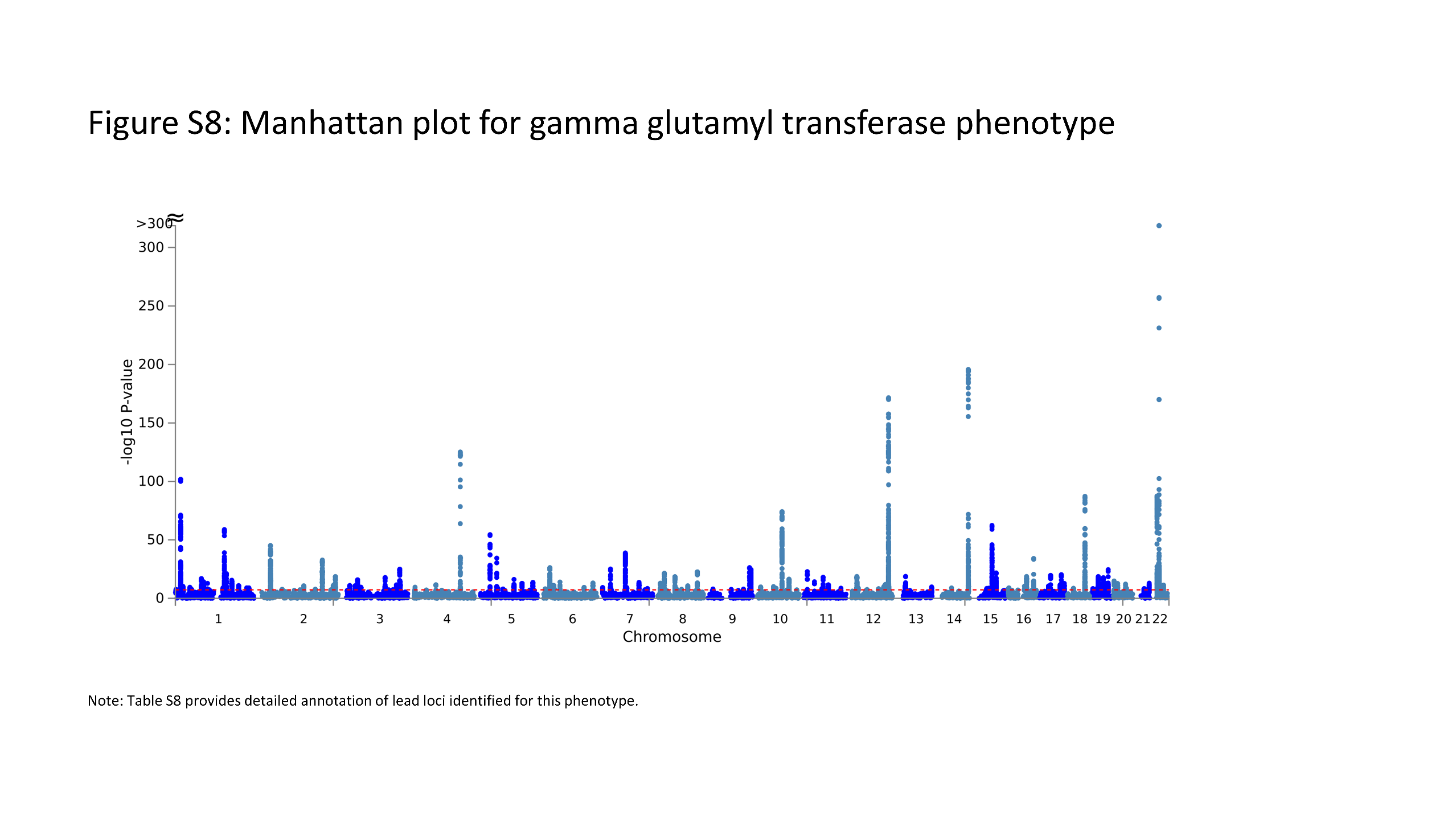


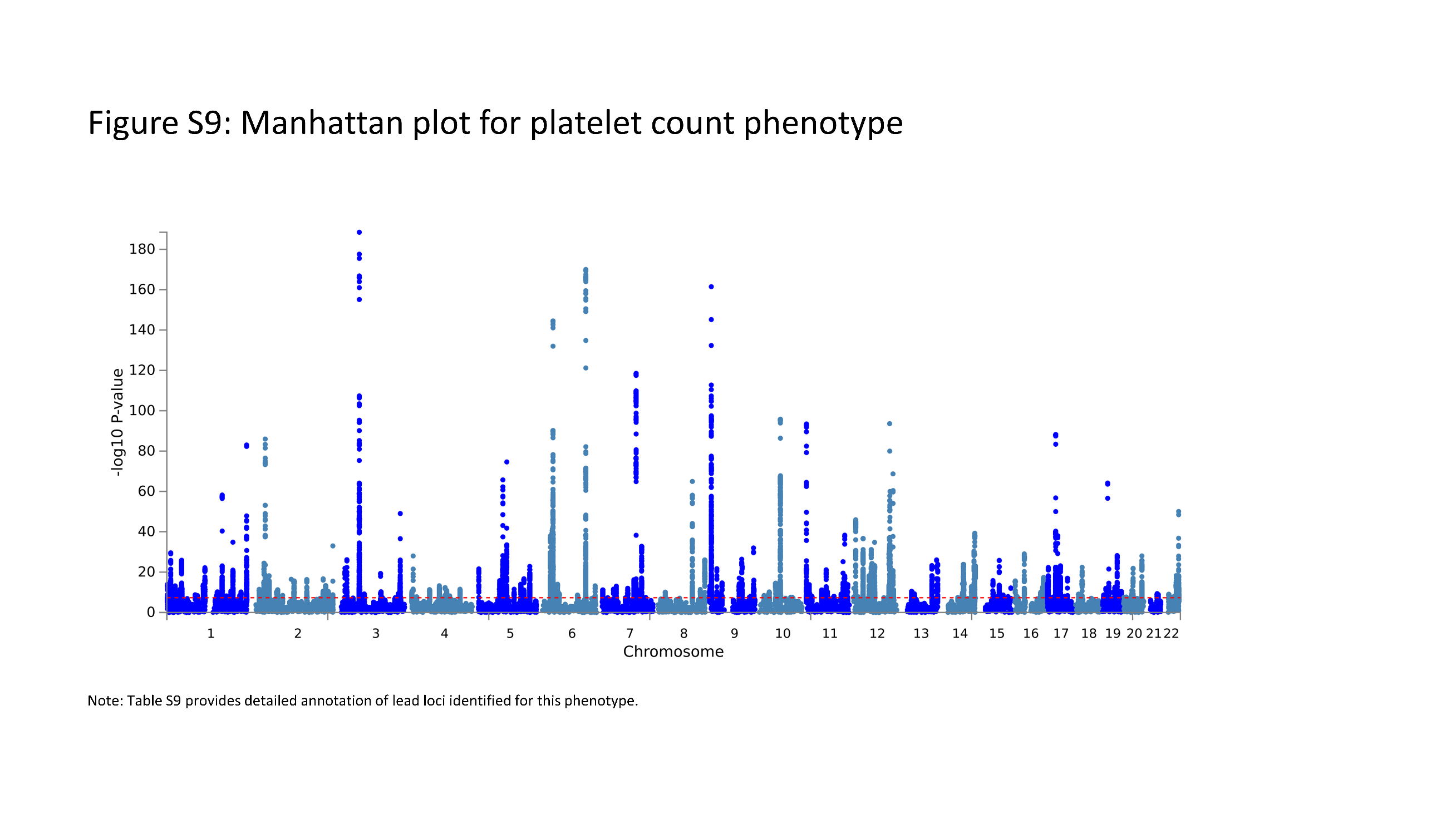


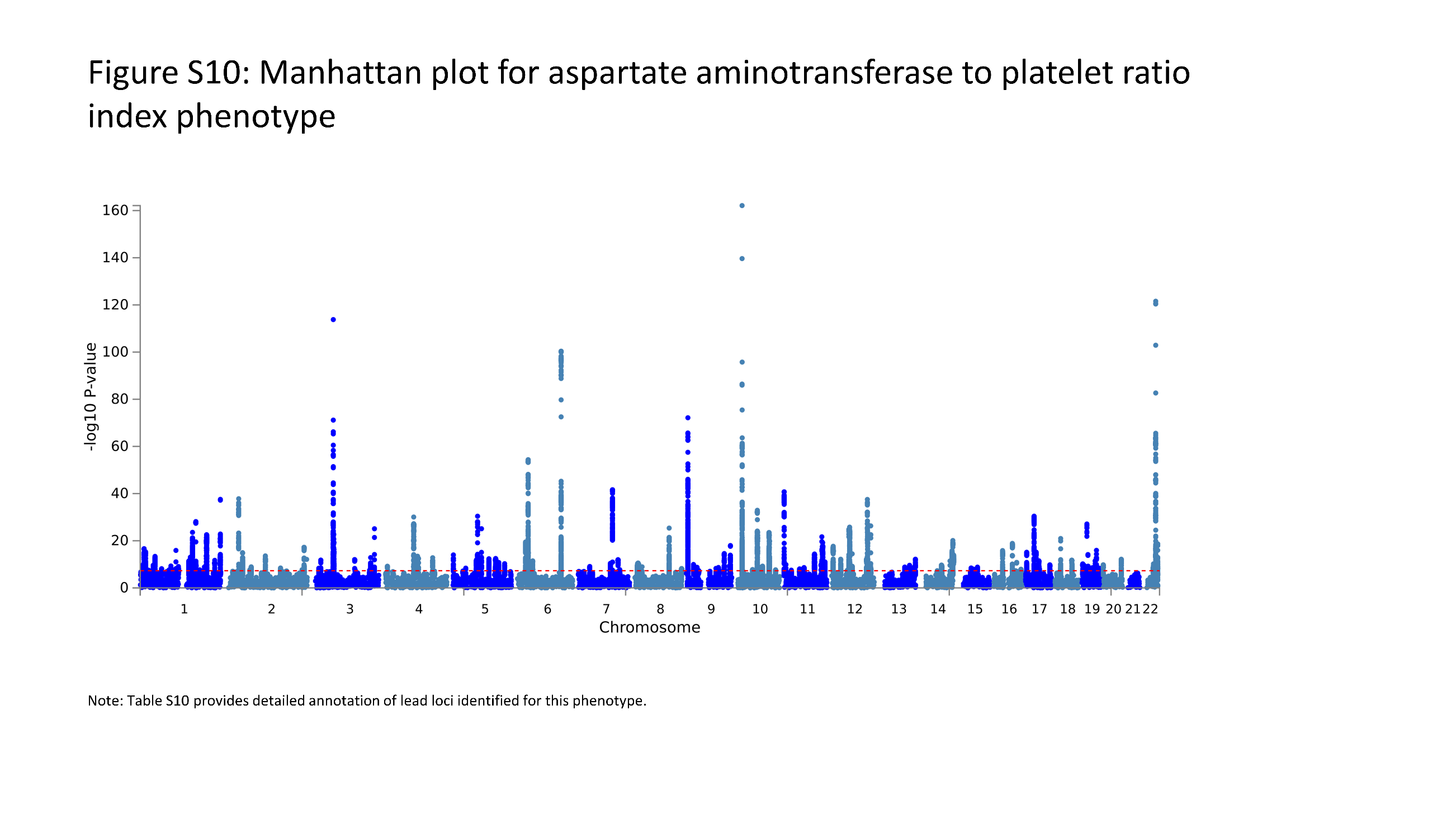


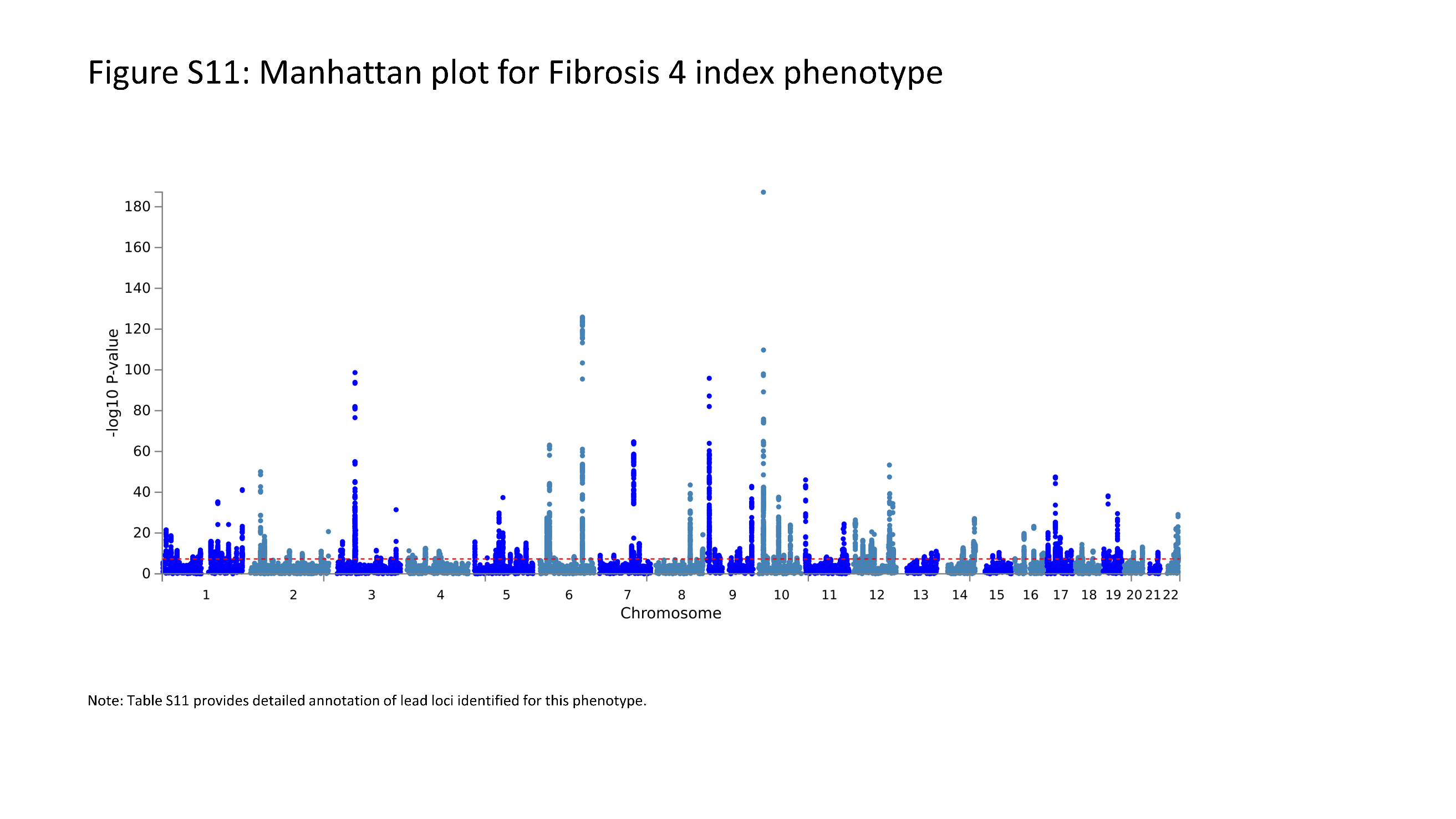


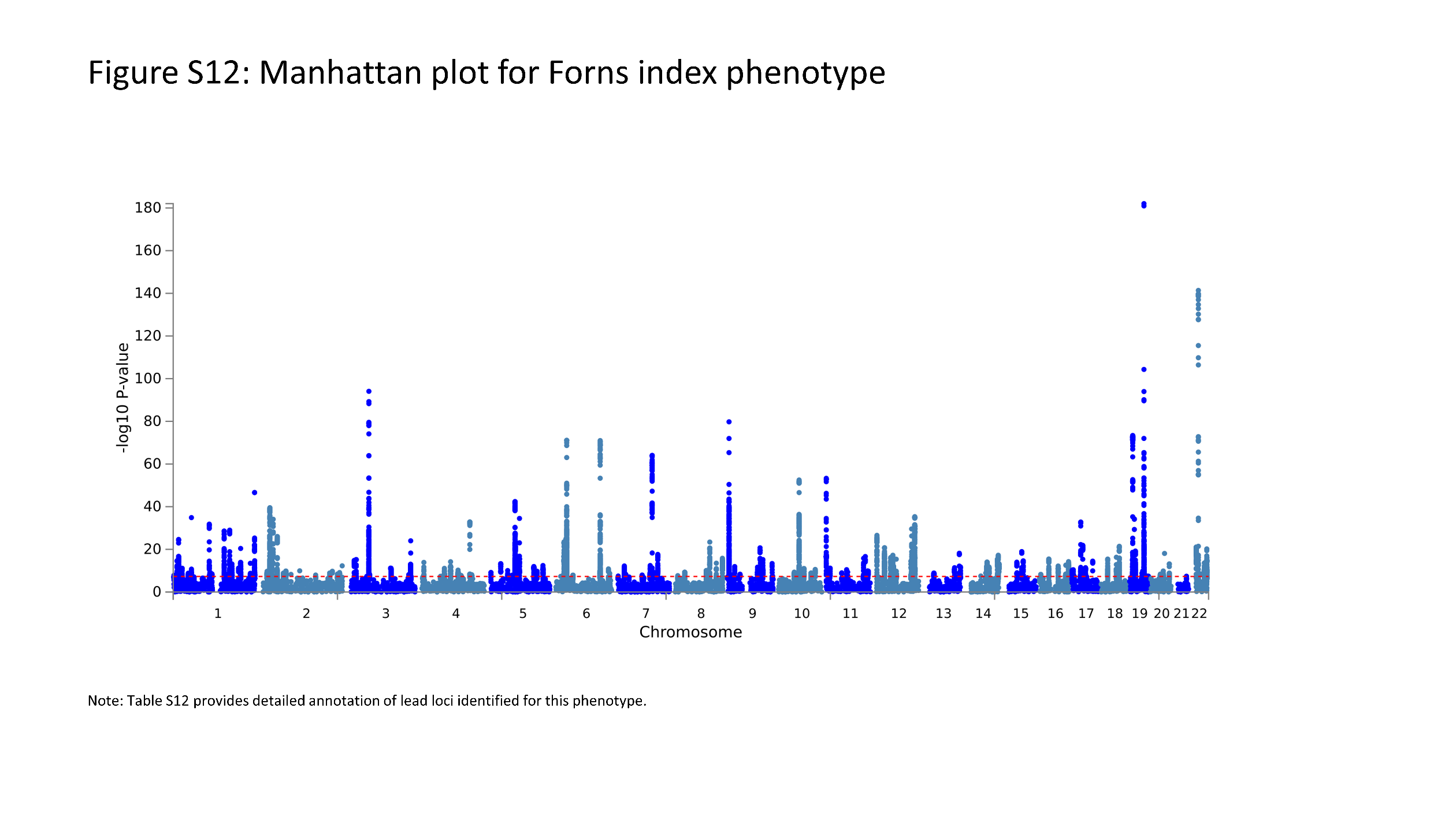


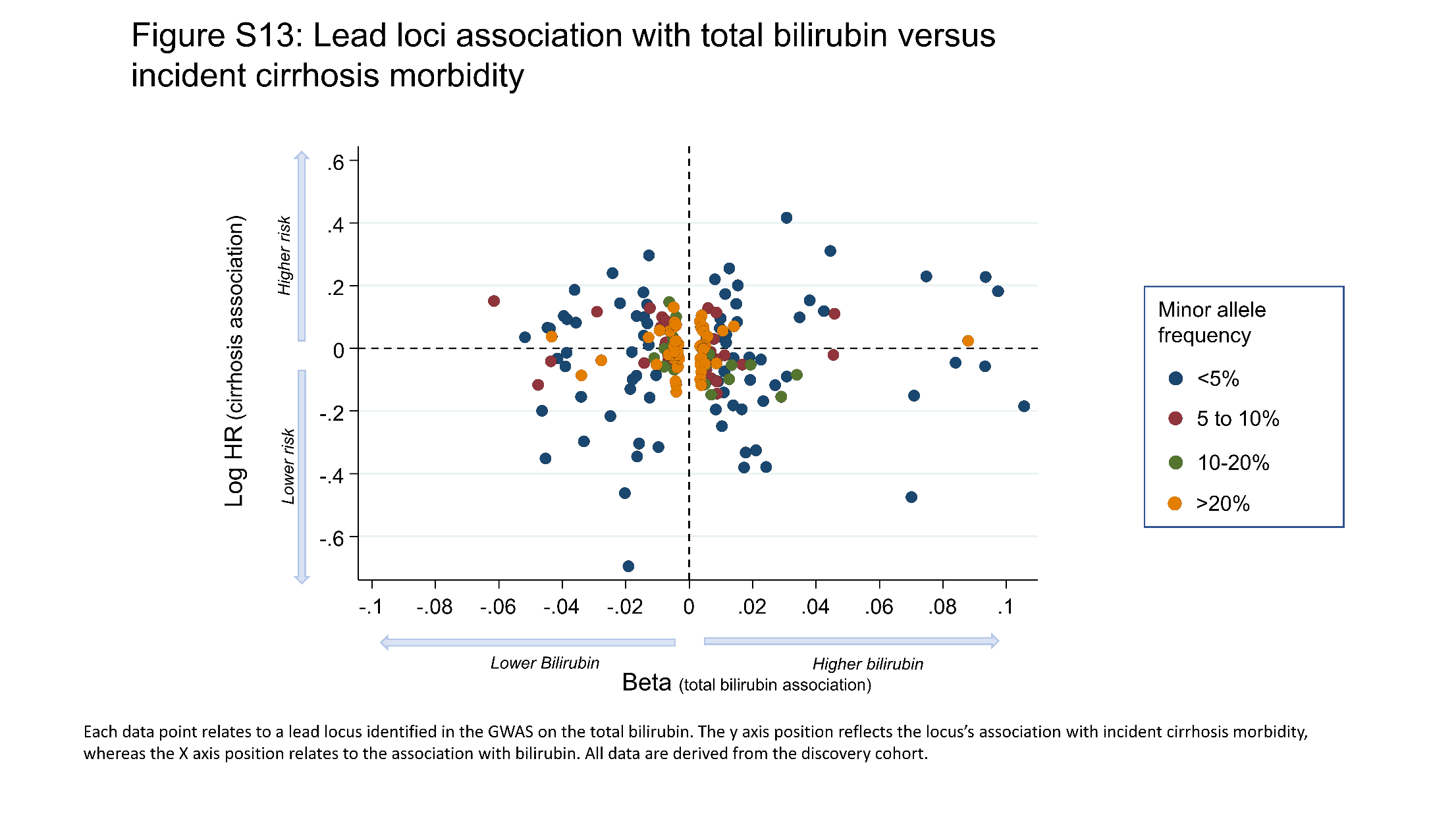


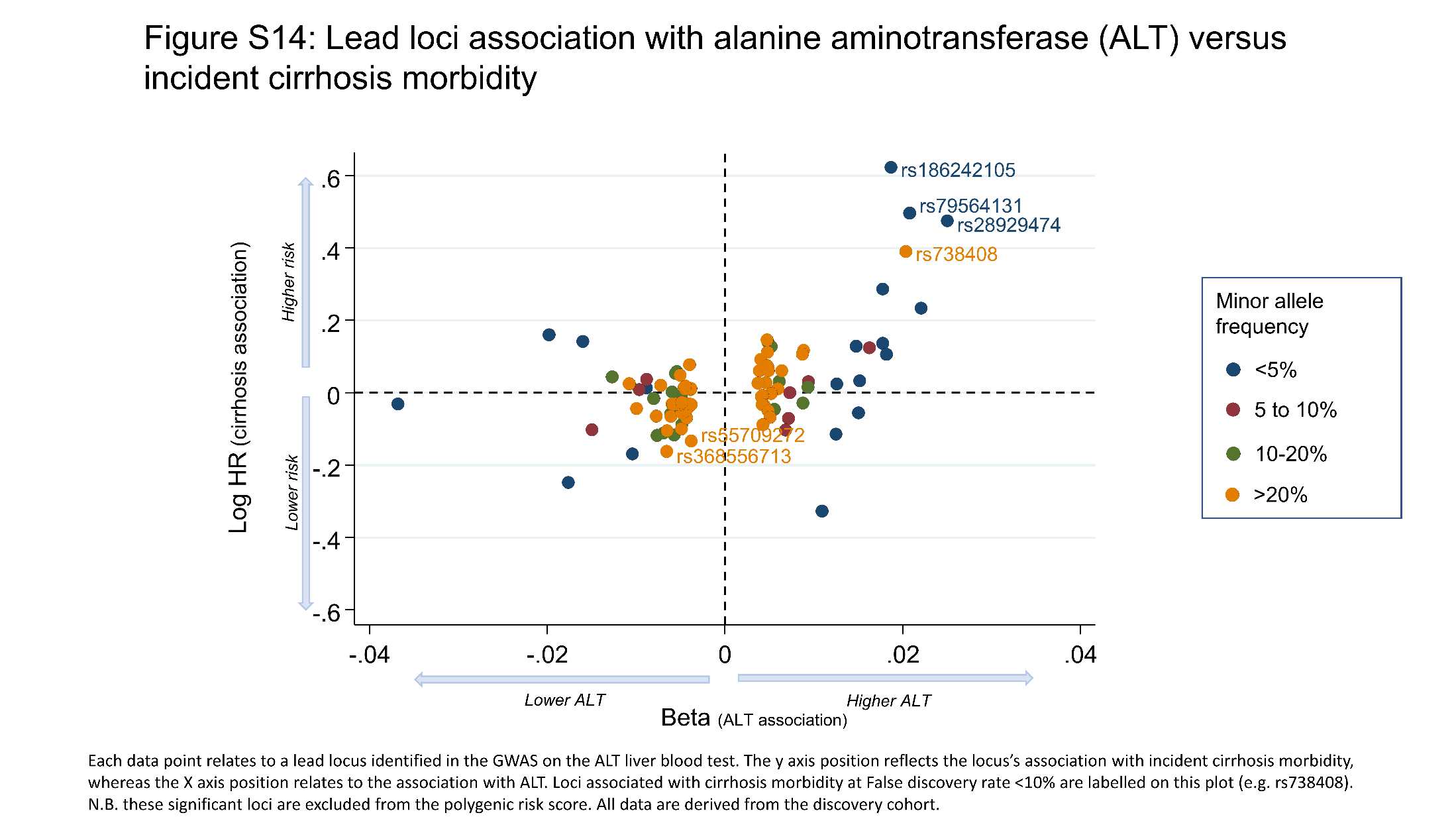


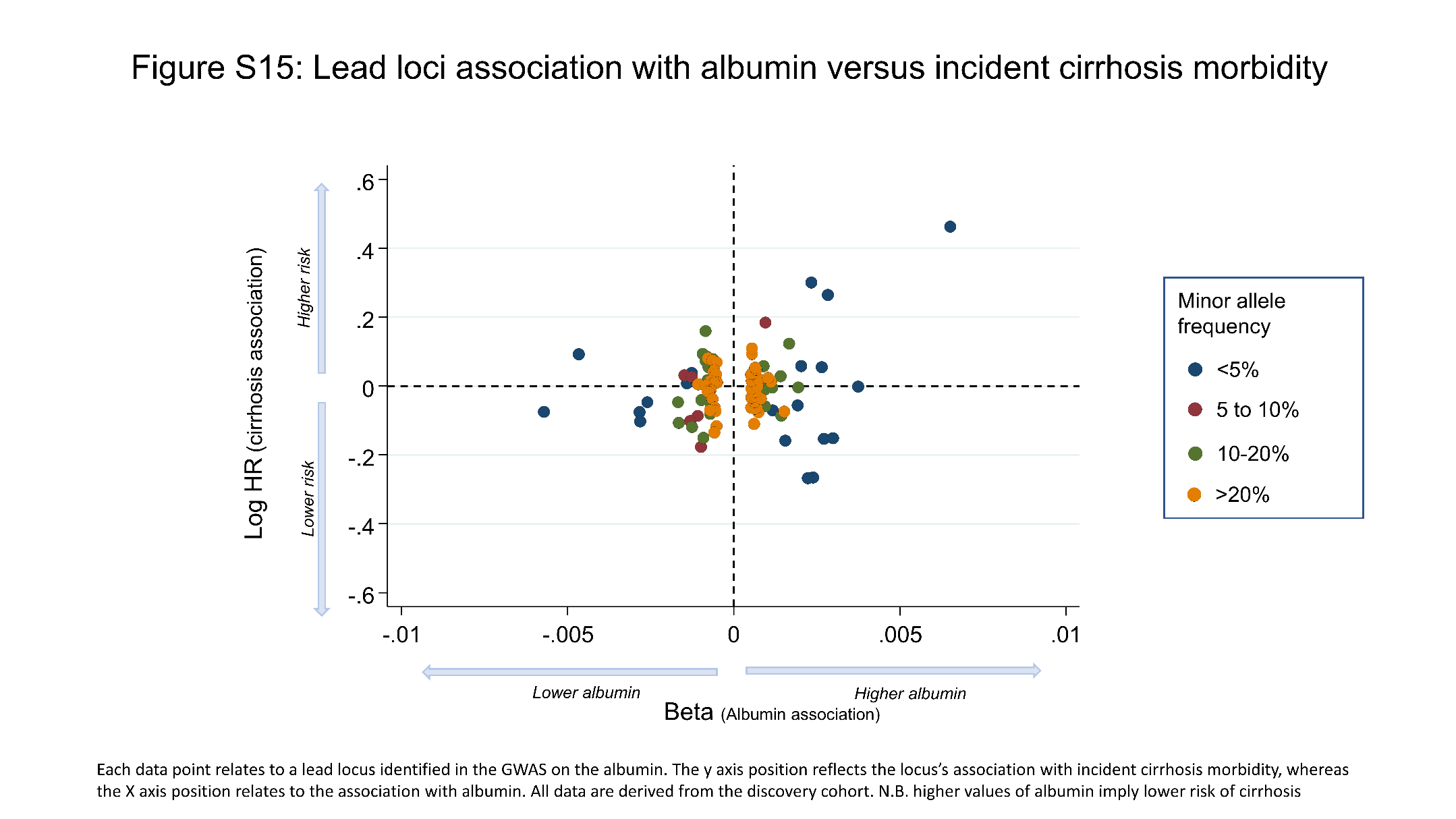


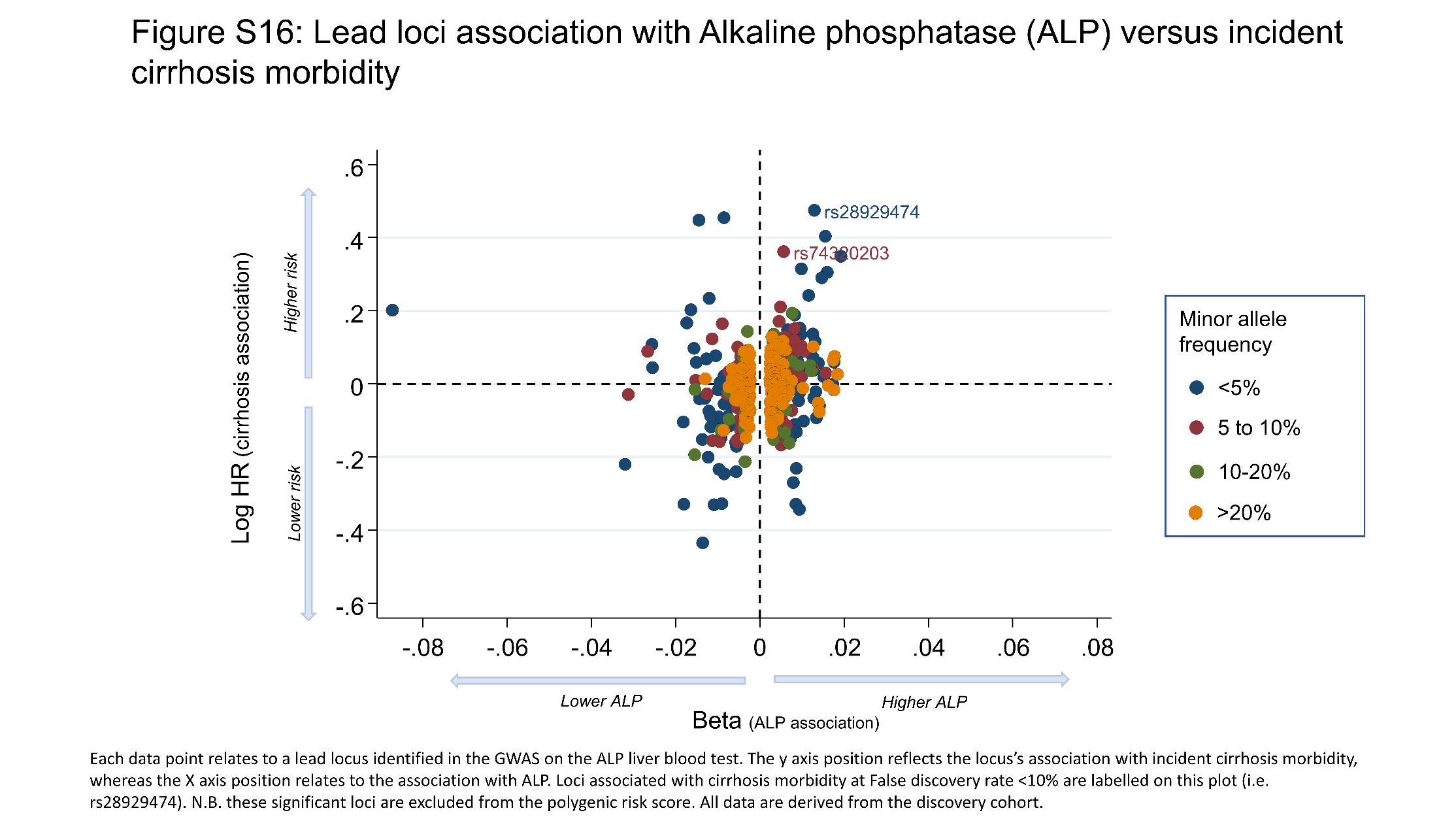


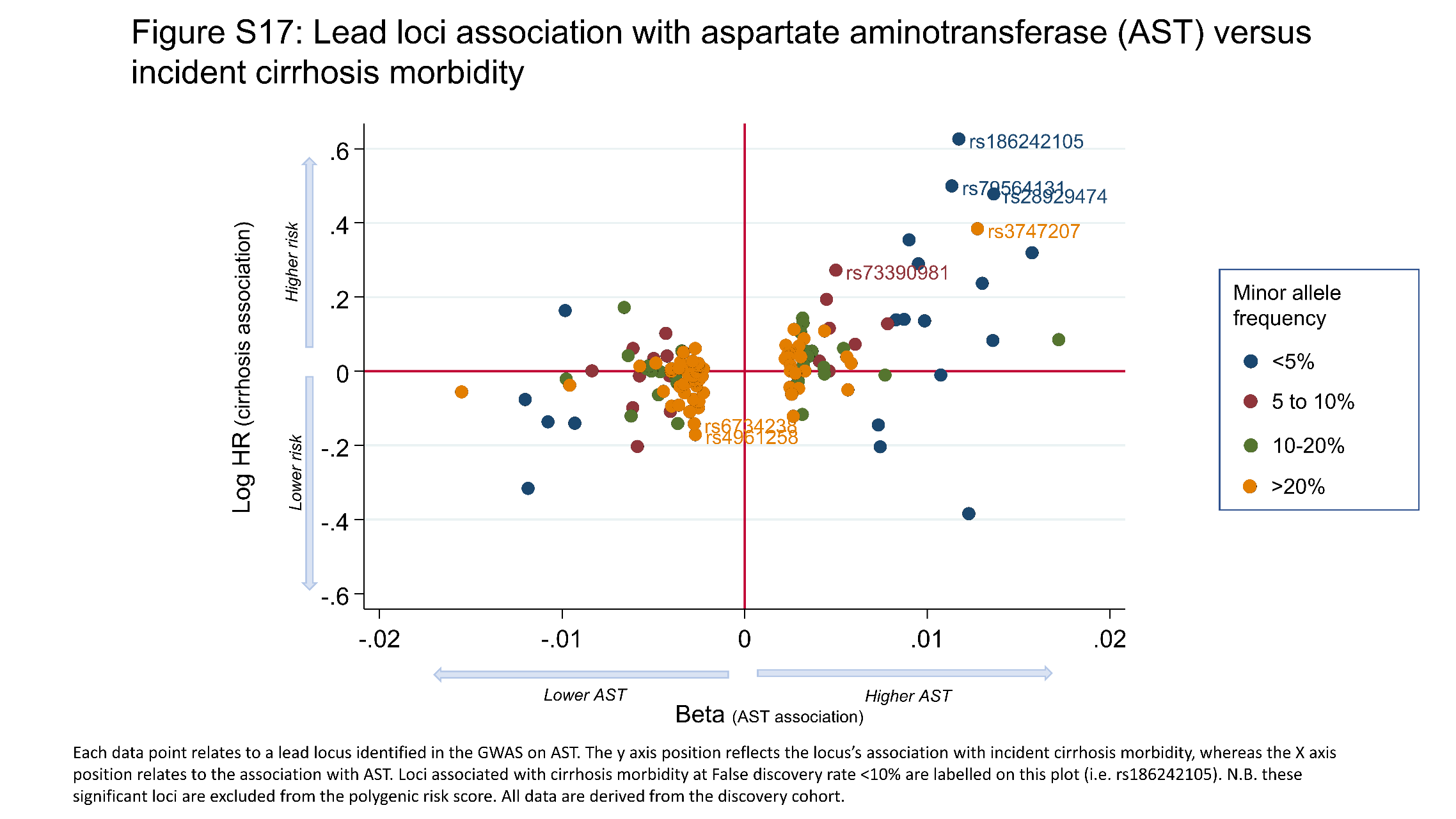


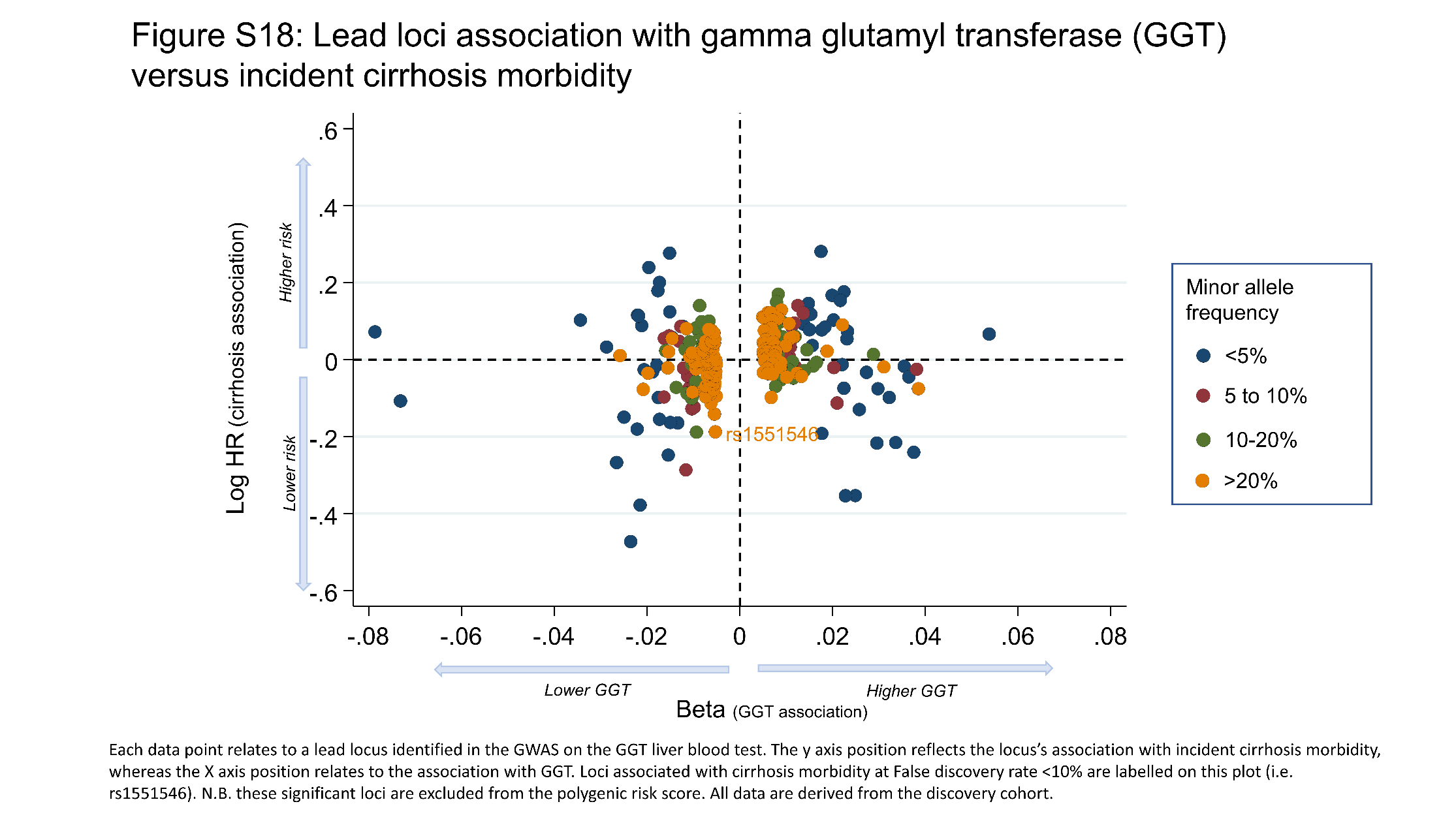


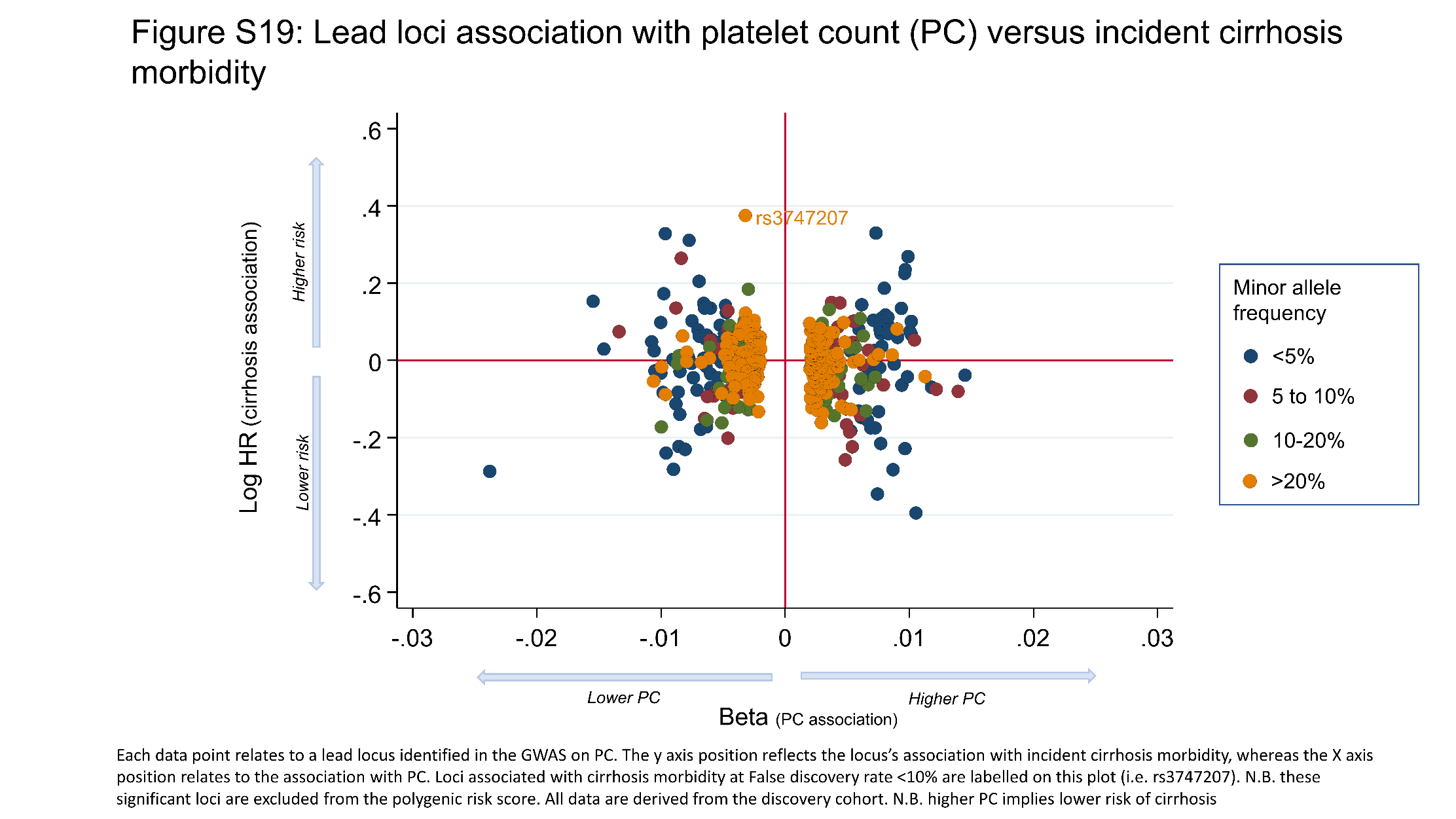


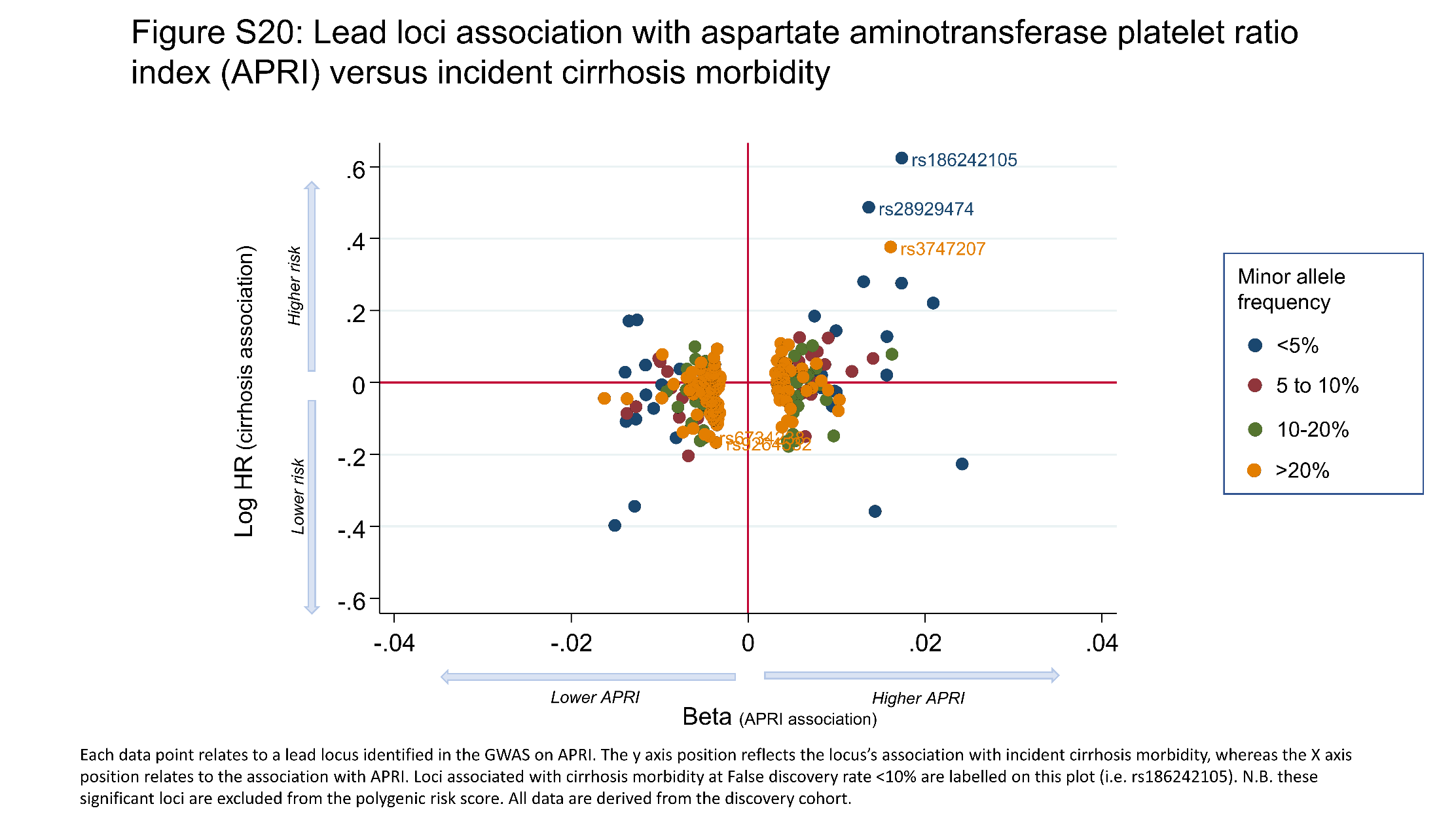


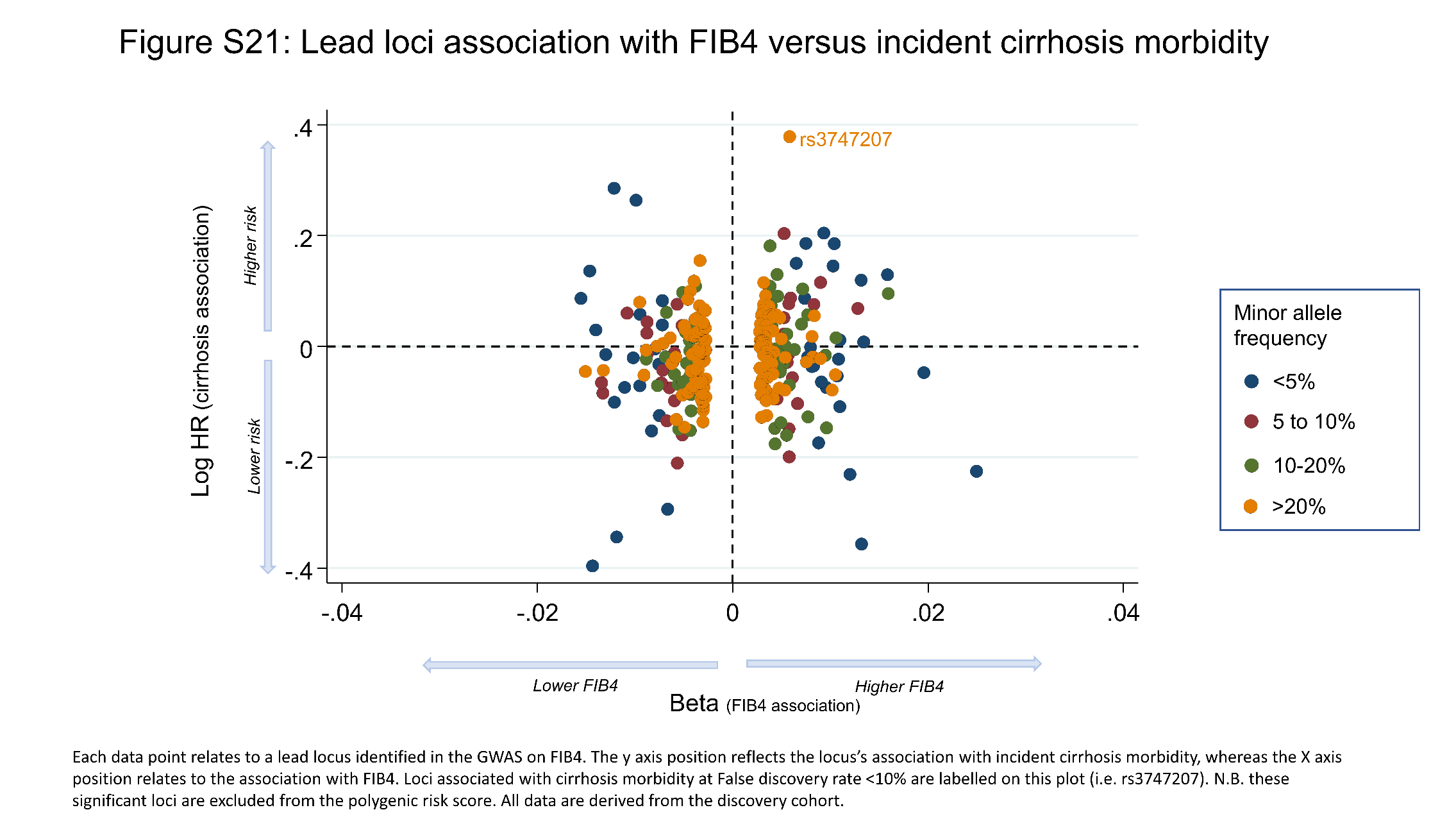


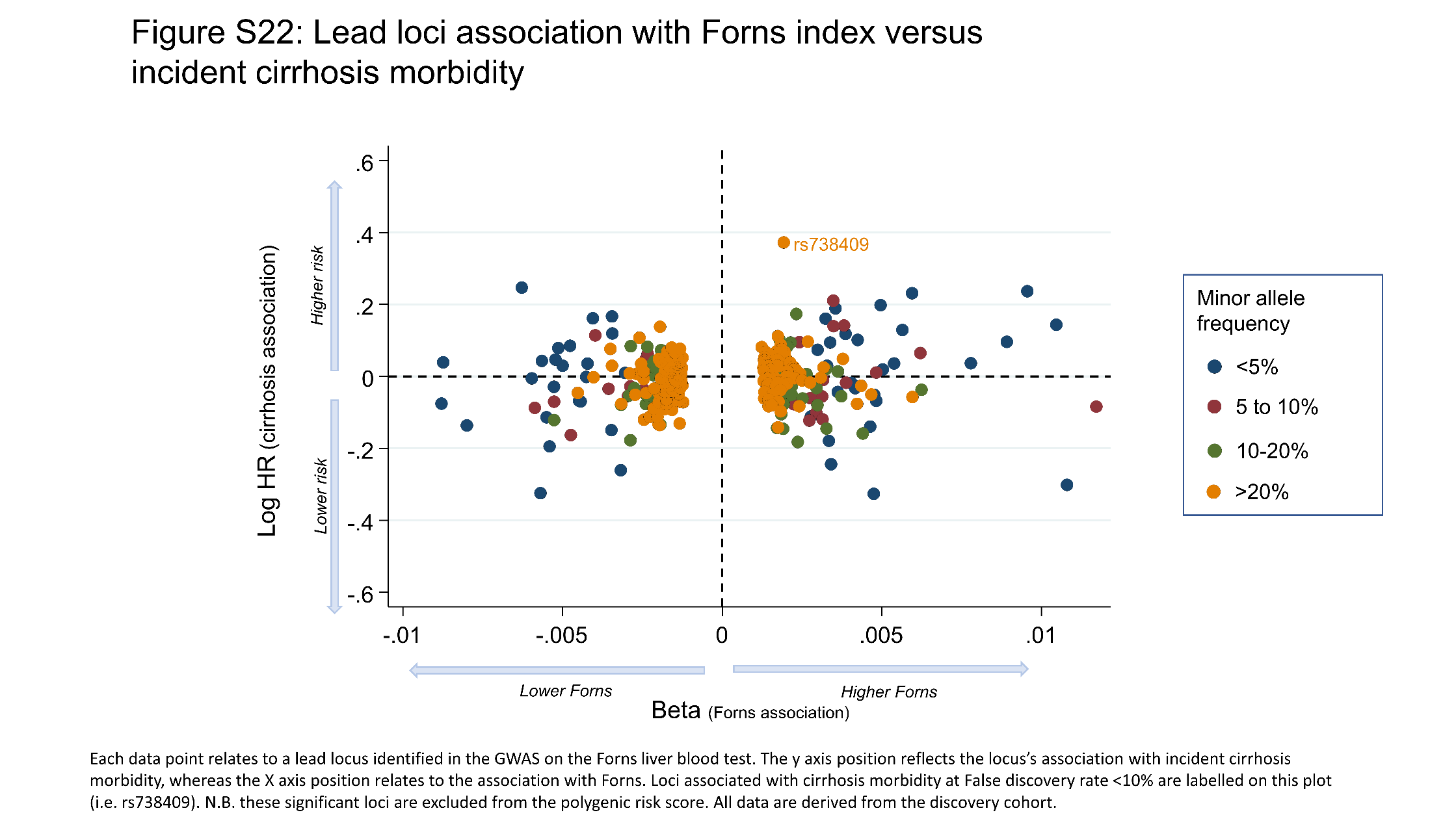

Supplement: Supplementary file 1 — Figure S1: Figure S2: Figure S3: Figure S4: Figure S5: Figure S6: Figure S7: Figure S8: Figure S9: Figure S10: Figure S11: Figure S12: Figure S13: Figure S14: Figure S15: Figure S16: Figure S17: Figure S18: Figure S19: Figure S20: Figure S21: Figure S22: [file LIV-44-3260-s002.docx]
